# Vaccinating children against influenza: overall cost-effective with potential for undesirable outcomes

**DOI:** 10.1186/s12916-019-1471-x

**Published:** 2020-01-14

**Authors:** Pieter T. de Boer, Jantien A. Backer, Albert Jan van Hoek, Jacco Wallinga

**Affiliations:** 1 Centre for Infectious Disease Control, National Institute for Public Health and the Environment, Antonie Van Leeuwenhoeklaan 9, 3721 MA Bilthoven, The Netherlands; 2 Department of Infectious Disease Epidemiology, Faculty of Epidemiology and Population Health, London School of Hygiene & Tropical Medicine, London, UK; 3 Department of Biomedical Data Sciences, Leiden University Medical Center, Leiden, The Netherlands

**Keywords:** Influenza, Vaccination, Dynamic transmission model, Cost-effectiveness, Economic evaluation, Children

## Abstract

**Background:**

The present study aims to assess the cost-effectiveness of an influenza vaccination program for children in the Netherlands. This requires an evaluation of the long-term impact of such a program on the burden of influenza across all age groups, using a transmission model that accounts for the seasonal variability in vaccine effectiveness and the shorter duration of protection following vaccination as compared to natural infection.

**Methods:**

We performed a cost-effectiveness analysis based on a stochastic dynamic transmission model that has been calibrated to reported GP visits with influenza-like illness in the Netherlands over 11 seasons (2003/2004 to 2014/2015). We analyzed the costs and effects of extending the current program with vaccination of children aged 2–16 years at 50% coverage over 20 consecutive seasons. We measured the effects in quality-adjusted life-years (QALYs) and we adopted a societal perspective.

**Results:**

The childhood vaccination program is estimated to have an average incremental cost-effectiveness ratio (ICER) of €3944 per QALY gained and is cost-effective in the general population (across 1000 simulations; conventional Dutch threshold of €20,000 per QALY gained). The childhood vaccination program is not estimated to be cost-effective for the target-group itself with an average ICER of €57,054 per QALY gained. Uncertainty analyses reveal that these ICERs hide a wide range of outcomes. Even though introduction of a childhood vaccination program decreases the number of infections, it tends to lead to larger epidemics: in 23.3% of 1000 simulations, the childhood vaccination program results in an increase in seasons with a symptomatic attack rate larger than 5%, which is expected to cause serious strain on the health care system. In 6.4% of 1000 simulations, the childhood vaccination program leads to a net loss of QALYs. These findings are robust across different targeted age groups and vaccination coverages.

**Conclusions:**

Modeling indicates that childhood influenza vaccination is cost-effective in the Netherlands. However, childhood influenza vaccination is not cost-effective when only outcomes for the children themselves are considered. In approximately a quarter of the simulations, the introduction of a childhood vaccination program increases the frequency of seasons with a symptomatic attack rate larger than 5%. The possibility of an overall health loss cannot be excluded.

## Background

Many European countries, including the Netherlands, have influenza vaccination programs that target older adults and people with certain health conditions [1]. These programs aim to offer direct protection to those at highest risk of complications; hence, the benefits of vaccination occur among the vaccine recipients themselves. Some European countries, including the UK and Finland [2, 3], extended their influenza vaccination programs to healthy children. Targeting children is expected to reduce the spread of influenza, offering indirect protection to unvaccinated individuals and non-effectively vaccinated high-risk individuals [4].

Dynamic transmission models are a useful tool to assess the expected level of direct and indirect protection that is provided by a vaccination program and to inform cost-effectiveness analyses of vaccination strategies [5]. When applied to childhood influenza vaccination programs, such dynamic transmission models need to capture the long-term infection dynamics due to changes in the proportion of immunes through vaccination and natural infection. They also need to account for the seasonal variability in vaccine effectiveness and in epidemic size due to the variation in vaccine match and antigenic drift. Dynamic transmission modeling studies were used to inform the decision to introduce an influenza vaccination program in the UK in 2013 for healthy children aged 2–16 years using the intranasally administered live-attenuated influenza vaccine (LAIV) [6]. The dynamic transmission models that were available at that time captured either the long-term infection dynamics [7] or seasonal variability in vaccine effectiveness and epidemic size [8], but not both.

A recently published stochastic transmission model that accounts for both the long-term infection dynamics and seasonal variability in vaccine effectiveness and epidemic size demonstrated that introduction of a childhood influenza vaccination program could lead to seasons with larger influenza epidemics, while, on average, reducing influenza incidence in the overall population [9, 10]. These large epidemics occur after a large proportion of susceptible individuals have accumulated over previous seasons with small epidemics. This transmission model projects a smaller decrease in influenza incidence after introducing childhood influenza vaccination programs as compared to models that only account for long-term infection dynamics [11, 12] or that only account for seasonal variability in vaccine effectiveness and epidemic size [8, 13].

Here, we performed a cost-effectiveness analysis of childhood influenza vaccination for the Netherlands that accounts for long-term infection dynamics as well as for the variability in vaccine effectiveness and epidemic size. We assessed the uncertainty in the health-economic outcome by conducting a probabilistic sensitivity analysis and using 1000 simulation runs with the stochastic transmission model. We assess the risk of undesirable outcomes, such as a decrease of health or an increase in the number of severe influenza seasons after introduction of the influenza vaccination program for children.

## Methods

### Model overview

Influenza transmission was simulated using a stochastic compartmental model to calculate seasonal infection attack rates by age group (in years) and risk group (presence of certain co-morbidities). Susceptibility and immunity to infection are defined relative to the circulating strains. Each season, an influenza epidemic unfolds according to an SIR model; the proportion of immunes increases due to infection or vaccination, and it decreases following immunity losses due to antigenic drift (more details are provided in Additional file 1). The model was calibrated to vaccine effectiveness data from the literature and data on laboratory-confirmed influenza cases in the Netherlands over the years 2003–2015 and to the reported numbers of visits to a general practitioner (GP) with influenza-like-illness in a Dutch sentinel surveillance network over 11 seasons (2003/2004–2014/2015) [10].

The calibrated transmission model was used to study the number of infections, clinical outcomes, and cost-effectiveness of the existing program for older adults and individuals with clinical risk conditions as compared to such a program that is extended with vaccination of healthy children. The childhood vaccination program was introduced in the year 2020. We considered the first 5 years after introduction as a stabilization period to allow adaptation of pre-existing immunity levels to the extended vaccination program and used the results of the seasons 2025–2026 to 2044–2045 for our analysis; the time horizon of our analysis was 20 seasons. Simulated outcomes on infections were converted to symptomatic cases, GP visits, hospitalizations, and deaths using age-specific and risk-group-specific outcome probabilities. Subsequently, clinical outcomes served as an input for the economic analysis estimating costs and loss of quality-adjusted life-years (QALYs). As recommended in the Dutch cost-effectiveness guideline [14], the analysis was conducted from a societal perspective. Future costs and QALYs were discounted to the value of the first recorded season of 2025–2026 using annual discount rates of 4% and 1.5%, respectively [14].

### Vaccination policies

The current Dutch influenza vaccination program offers free annual vaccination to all persons aged ≥ 60 years and individuals aged < 60 years with certain health conditions using the trivalent inactivated vaccine (TIV). Vaccination coverage by age group and risk group of the current program was obtained from the literature (approximately 21% of the total population; 2.9% of < 20-year-olds, 8.7% of 20–59-year-olds and 65.9% of ≥ 60-year-olds) [15] and was assumed to remain constant over time. The additional vaccination program for healthy children consists of annual vaccinations for children aged 2–16 years using the intranasally administered quadrivalent LAIV (Q-LAIV). This age-group covers children attending day care, primary school, or secondary school. A lower limit of 2 years was used because Q-LAIV is contraindicated in children younger than this age [16]. The coverage in healthy children was assumed at 50%; this approximates the average uptake of the Q-LAIV program in England during the 2017/2018 season, being 43% in children aged 2–3 years at their GP and 60% in children aged 4–9 years at schools [17, 18]. We assumed consistent vaccination in healthy children, i.e., the same children are vaccinated every season.

### Model input

#### Transmission model

The model input of the dynamic transmission model is available in Additional file 1: Table S1 and in [9, 10]. Briefly, the transmission model was informed by Dutch demographic projections from 2015 to 2044 [19], and social contact structures by age and sex class were based on observed contact patterns in the Netherlands [20]. The duration of protection through natural infection is measured with respect to the circulating influenza strains. This parameter was estimated in the model calibration procedure and lasted on average 5.1 years (95% interval 2.9–8.2 years) [9]. Vaccine effectiveness of TIV against the circulating influenza strains was on average 45% (95% interval 19–66%), and simulated vaccine matches varied by season and decreased with age [21–23]. Recent post-licensure effectiveness studies comparing the effectiveness between LAIV and the inactivated influenza vaccine found equivocal results [24–26], while a clustered randomized trial found no difference between LAIV and inactivated influenza vaccine on a community level [27]. Therefore, we assumed a similar effectiveness of Q-LAIV and TIV. As the vaccine effectiveness of TIV wanes already within the season, we assumed its duration of protection to last 1 year. No long-term effectiveness data is available for Q-LAIV, but a clinical trial in young children suggests that the vaccine could provide some protection in the second season after vaccination [28]. As live-attenuated vaccines are thought to be less immunogenic than natural infections due to a lower antigen load [29], we assumed in the main analysis that the duration of protection through Q-LAIV lasts 1 year and explored a longer duration of protection in the sensitivity analysis.

#### Outcome probabilities

Probability of symptomatic infection given infection and subsequent GP visit were obtained from the literature [30, 31]. Influenza-related mortality and hospitalization rates by age group and risk group were estimated using the fraction of all-cause deaths that were associated with influenza [32], proportions of deaths in the hospital [33], and hospitalization-fatality rates (see Additional file 1: Table S2) [34].

#### Quality-adjusted life-years

QALY losses due to influenza illness were based on the literature (see Additional file 1: Table S3). QALY losses due to influenza-associated premature death were estimated using the life expectancy at age of death and quality-of-life population norms by age [35]. To account for the increase in life expectancy over time, we used cohort life tables. Projections of age-specific probabilities of death were used to estimate for each cohort the risk of dying over the course of their lifetime [19]. Projected probabilities of death were available up to 2060, and we assumed these to be fixed thereafter.

#### Costs

Costs from earlier years were converted to 2017 using the Dutch consumer price index [36]. The total cost per administered dose was €14.95, including a vaccine cost of €3.59 [37] and an administration cost of €11.36 [38]. The vaccine cost was based on the current price of TIV in a public program, while the administration cost is the current fee a GP receives for the patient invitation, vaccine administration, vaccine storage, and disposal of waste and unused vaccines. No costs of adverse event of Q-LAIV were included as these have been reported to be mild, short-lasting, and transient. Costs related to influenza illnesses were estimated using data from the literature and other Dutch data sources (see Additional file 1: Table S3). Direct healthcare costs of influenza include costs related to GP visits, which includes also prescribed medication and referrals to the specialist, and hospitalization costs.

According to the most recent Dutch guideline on cost-effectiveness research [14], indirect healthcare costs (i.e., health care costs unrelated to influenza in life-years gained) should also be taken into account. These costs were estimated using the remaining life-expectancy at age of death and annual age-specific healthcare cost unrelated to influenza or pneumonia from a specifically developed tool [39]. Patient costs include cost due to over-the-counter medication and travel cost. Productivity losses included costs due to work absence from paid work of sick people themselves (15–69 years) or caregivers of a sick child (< 15 years). Productivity losses of premature deaths were valued using the friction method [40], assuming that the work absence was limited to a friction period of 85 days [14].

### Cost-effectiveness analysis

The stochastic transmission model generated 1000 simulated series of 20 consecutive seasons. For each simulation, a set of health-economic parameter inputs was sampled from distributions as specified in Additional file 1: Table S3. Next, discounted costs and QALY losses over the analyzed period of 20 years were summed up and averaged across simulations. The incremental cost-effectiveness ratio (ICER) was calculated by dividing the difference in costs between two strategies by the difference in QALYs. The childhood vaccination program was considered cost-effective when the ICER was below €20,000 per QALY gained, the conventional Dutch threshold for preventive interventions [41].

### Sensitivity analysis

For each individual simulation, the net costs and net QALYs relative to the current vaccination program were plotted in a cost-effectiveness plane. The probability of the optimum policy over a range of cost-effectiveness thresholds was plotted in a cost-effectiveness acceptability curve. In a univariate sensitivity analysis, we explored variation of the targeted age-group (2–3 years and 2–12 years), coverage in healthy children (25% and 75%), duration of protection of Q-LAIV (2 years and 5 years), efficacy of Q-LAIV (50% higher efficacy than TIV or full protection), vaccine price, cost components considered, and QALY losses due to influenza.

### Number of seasons with large influenza epidemics

We investigated the variability in epidemic size by monitoring the number of seasons with large epidemics. We defined a large epidemic as a season with a symptomatic infection attack rate larger than 5%. This threshold was based on the symptomatic attack rate in the 2017/2018 season in the Netherlands [42]. In this season, hospitals reported capacity issues due to large numbers of severely ill patients and high sick leave among healthcare workers.

## Results

### Clinical impact

Table 1 shows the 20-year average annual number of clinical events in the Netherlands for each vaccination alternative. Under the current vaccination program, we estimated across 1000 simulations on average 317,703 (95% interval 239,785–391,519) symptomatic cases; 71,552 (53,140–89,952) GP visits; 7703 (4960–10,391) hospitalizations; and 3234 (2057–4475) deaths per year. Extending the current program with vaccination of children aged 2–16 years at 50% coverage would prevent 57,266 (95% interval 19,116–116,418) symptomatic cases; 13,352 (5113-25,873) GP visits; 1288 (547–2306) hospitalizations; and 318 (− 105–844) deaths per year. When only outcomes among children aged 2–16 years were included, the average reduction was estimated at 31,522 (21,676–43,348) symptomatic cases; 7628 (5550–10,111) GP visits; 615 (464–789) hospitalizations; and 0.5 (− 0.2 to 1.1) deaths per year. Hence, 55% of the averted symptomatic cases occurred in the target group, while this was 0.1% of the averted deaths.

**Table 1:** The predicted 20-year annual average number of clinical events in the Netherlands in the absence and presence of childhood influenza vaccination for children aged 2–16 years at 50% coverage. Events are shown for the entire population and for the targeted age group only

Outcome	Symptomatic cases		GP visits		Hospitalizations		Deaths	
	Mean (95% interval)^a^	Rate^b^	Mean (95% interval)^a^	Rate^b^	Mean (95% interval)^a^	Rate^b^	Mean (95% interval)^a^	Rate^b^
Within the general population								
CP	317,703 (239,785–391,519)	1781	71,552 (53,140–89,952)	401	7703 (4960–10,391)	43.1	3234 (2057–4475)	18.1
CP + 2–16 y	260,437 (123,289–354,634)	1460	58,200 (26,929–79,538)	326	6415 (2749–9484)	35.9	2916 (1208–4353)	16.3
Reduction	57,266 (19,116–116,418)	321	13,352 (5113–25,873)	75	1288 (547–2306)	7.2	318 (− 105 to 844)	1.8
Within children aged 2–16 y								
CP	86,779 (69,895–102,394)	3018	20,546 (15,968–24,684)	714	1480 (934–2018)	51.5	1.6 (.8–2.5)	0.054
CP + 2–16 y	55,257 (28,278–72,633)	1921	12,918 (6428–17,287)	449	865 (371–1284)	30.1	1.1 (.4–1.9)	0.038
Reduction	31,522 (21,676–43,348)	1097	7628 (5550–10,111)	265	615 (464–789)	21.4	0.5 (− 0.2 to 1.1)	0.016

^a^Based on 1000 simulations, 95% interval uses the 2.5% and 97.5% percentiles. ^b^Rate per 100,000 population. *CP* current program, *y* years

### Cost-effectiveness

Table 2 shows the 20-year cumulative economic impact and cost-effectiveness of vaccination of children aged 2–16 years at 50% coverage in the Netherlands. Across 1000 simulations, the childhood vaccination program resulted in an average gain of 43,525 QALYs, of which 90% was due to the prevention of mortality. However, the childhood vaccination program increased the total costs by €172 million. When stratified by cost component, vaccination costs increased by on average €286 million and indirect healthcare costs increased by on average €344 million, while the direct healthcare costs decrease by on average €69 million, patient costs decreased by on average €86 million, and productivity losses decreased by on average €303 million. Dividing the average net cost by the average number of QALYs saved resulted in an ICER of €3944 per QALY gained.

**Table 2:** The 20-year cumulative impact and cost-effectiveness of vaccination of children aged 2–16 years at 50% coverage in the Netherlands. Events are shown for the entire population and for the targeted age group only. Outcomes are averaged over 1000 simulations. QALY losses and costs include an annual discount rate of 1.5% and 4%, respectively. *CP* Current program, *HC* Healthcare, *y* years

Outcome	Within the general population			Within children aged 2–16 y		
	CP	CP + 2–16 y	Difference	CP	CP + 2–16 y	Difference
QALY loss (thousands)						
QALY loss illness	23	19	− 4	6.2	3.9	− 2.3
QALY loss mortality	410	370	− 39	1.1	0.8	− 0.3
Total QALY loss	433	389	− 44	7.3	4.7	− 2.6
Costs (€, millions)						
Vaccination	964	1250	286	17	303	286
Direct HC costs	467	398	− 69	65	39	− 26
Indirect HC costs	0	344	344	0	0	0
Patient costs	482	396	− 86	128	82	− 47
Productivity loss	2214	1911	− 303	179	115	− 64
Total costs	4127	4299	172	390	539	149
Cost-effectiveness						
ICER (€/QALY gained)			3944			57,054

*CP* current program, *HC* healthcare, *QALY* quality-adjusted life-year

When only outcomes among children aged 2–16 years were considered, the childhood vaccination program resulted in an average gain of 2611 QALYs, of which 13% was due to the prevention of mortality. The average total costs were estimated to increase by €149 million, with most of the cost savings due to averted productivity losses among caregivers. The ICER then was estimated at €57,054 per QALY gained.

### Sensitivity and uncertainty analysis

#### Probabilistic sensitivity analysis

**Fig. 1:**
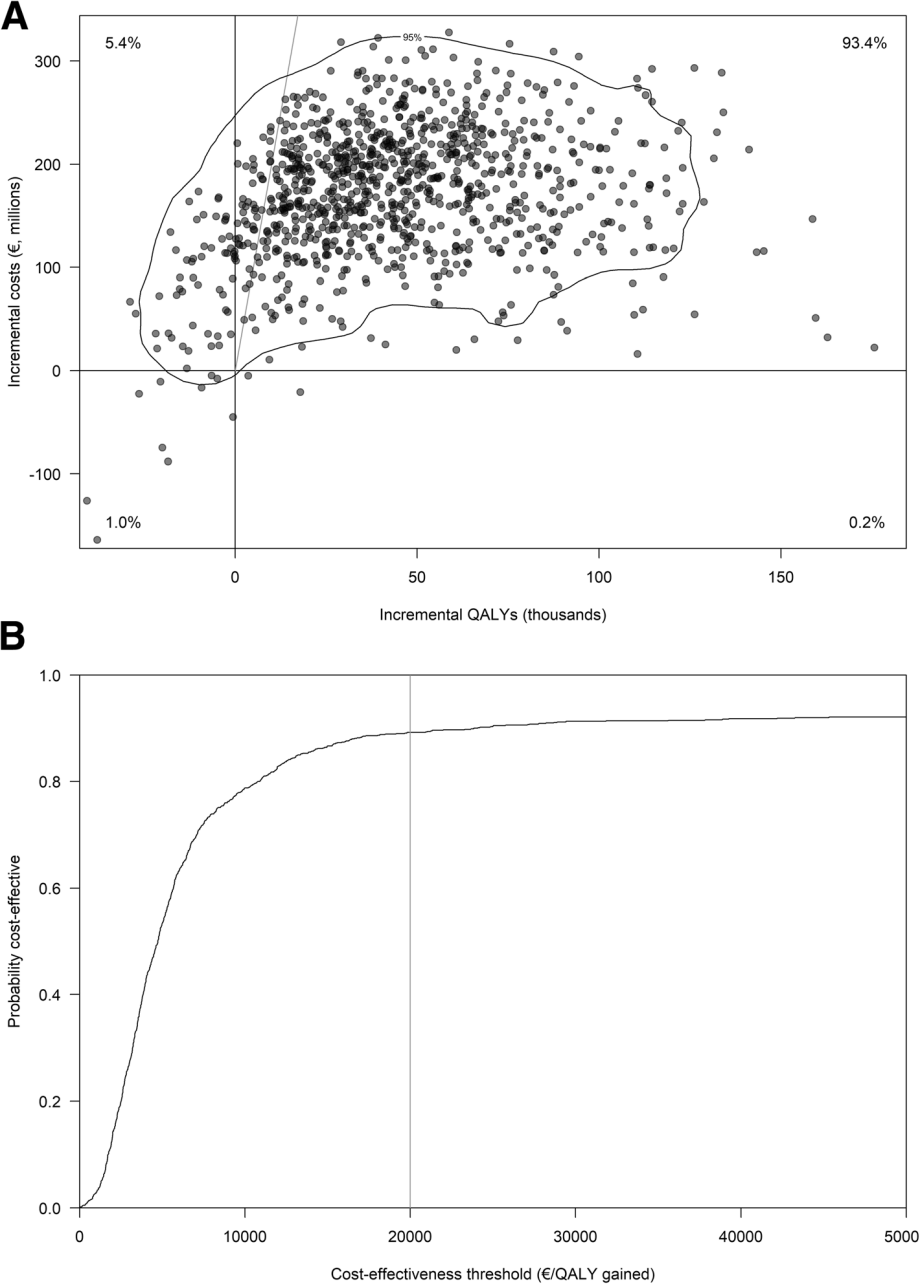
Probabilistic sensitivity analysis using 1000 simulations of extending the existing program with vaccination of children aged 2–16 years at 50% coverage in the Netherlands over 20 seasons. **a** The cost-effectiveness plane depicts the incremental costs and QALYs of the individual simulations. The contour line represents the 95% interval of the simulations. The gray line indicates the conventional Dutch cost-effectiveness threshold of €20,000 per QALY gained. **b** The cost-effectiveness acceptability curve depicts the proportion of cost-effective simulations over a range of cost-effectiveness thresholds

Figure 1 shows results of a probabilistic sensitivity analysis using 1000 simulations. The cost-effectiveness plane indicates that the uncertainty in the economic impact of the childhood vaccination program is substantial (Fig. 1a). Measured over 20 seasons, the childhood vaccination program resulted in a QALY gain against higher total costs in 93.4% of the simulations and a QALY gain against lower total costs in 0.2% of the simulations. The cost-effectiveness acceptability curve demonstrated that the childhood vaccination program was cost-effective for a threshold of €20,000 per QALY gained (Fig. 1b) in 89.3% of the simulations. However, in 6.4% of the simulations, the childhood influenza vaccination resulted in a net QALY loss.

When only outcomes among children aged 2–16 years were considered, the childhood vaccination program resulted in a QALY gain against higher total costs in all simulations (Additional file 2: Figure S1). At a threshold of €20,000 per QALY gained, the childhood vaccination program was cost-effective in 0.9% of the simulations. When the cost-effectiveness results are stratified in yearly age-groups, the highest net benefits were found among older adults and working adults, particularly the parental age group of 30–40 years, whereas the lowest net benefits are in the targeted children themselves (Additional file 2: Figure S2).

#### Univariate sensitivity analysis

**Fig. 2:**
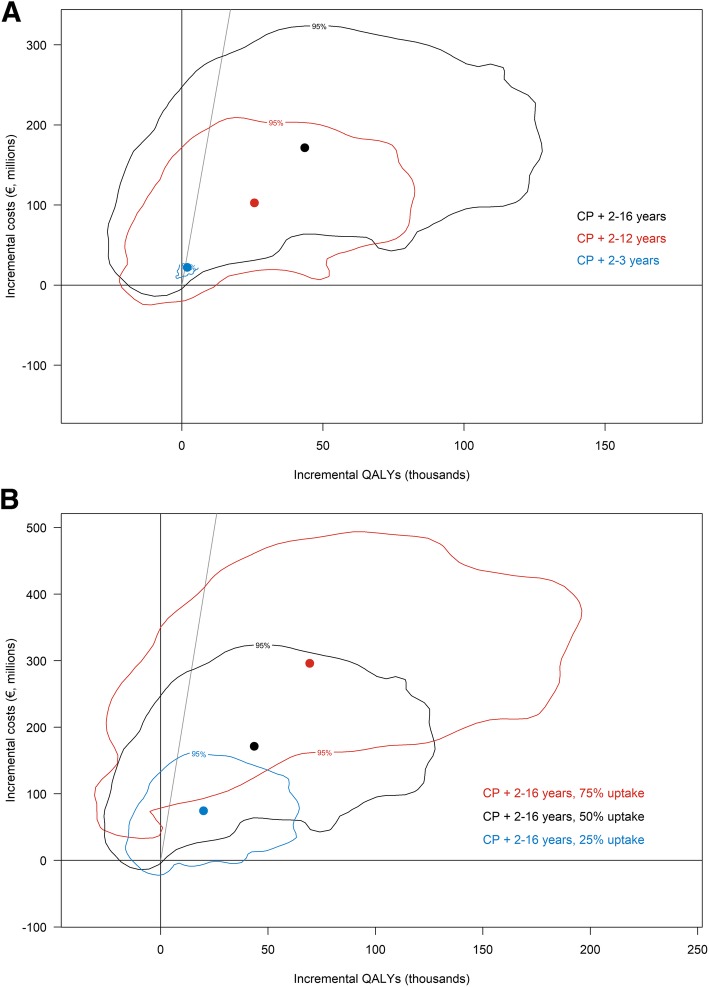
Univariate sensitivity analysis of the **a** targeted age-group of vaccination, and **b** the uptake rate in healthy children. The cost-effectiveness plane contains the incremental costs and quality-adjusted life-years (QALYs) of adding the childhood program to the current program (CP). The colored dots represent the average outcome, and the contour line represents the 95% interval of the simulations. The gray line indicates the conventional Dutch cost-effectiveness threshold of €20,000 per QALY gained

Figure 2 shows that ICER and the probability of a net QALY loss were not sensitive to the targeted age group or vaccination coverage (more details in Additional file 2: Tables S1, S2, S3, S4, S5, S6 and Figure S3). However, the average impact and the uncertainty of the economic outcomes increased when a larger age group was targeted or a higher coverage was assumed.

**Fig. 3:**
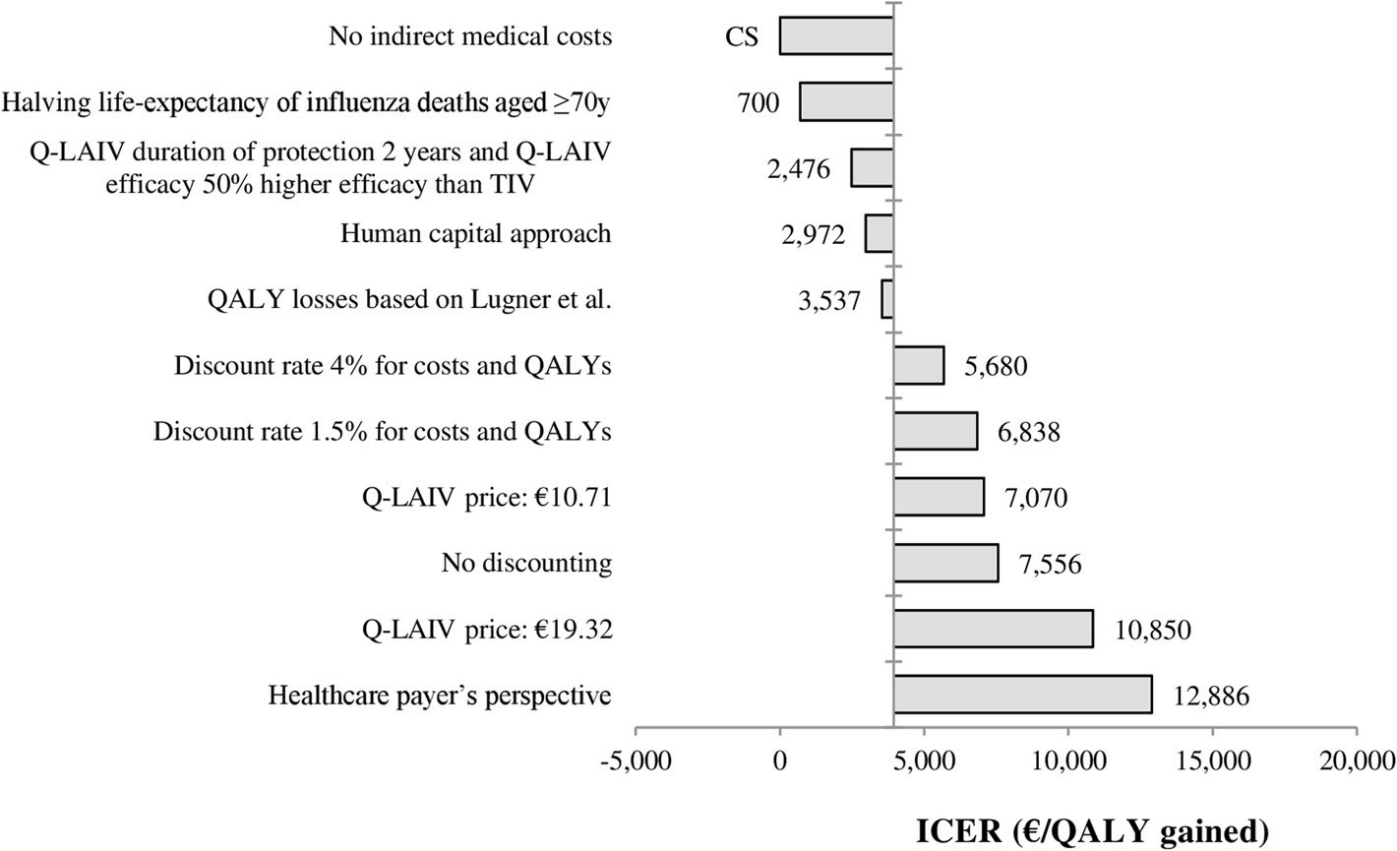
Univariate sensitivity analysis of extending the current program with vaccination of children aged 2–16 years at 50% coverage. The human capital approach values productivity losses of premature influenza deaths until the age of retirement. CS, cost-saving; ICER, incremental cost-effectiveness ratio; QALY, quality-adjusted life-year; Q-LAIV, quadrivalent live-attenuated influenza vaccine; y, years

Increasing the duration of protection of Q-LAIV from 1 to 2 years or to 5 years or increasing the vaccine efficacy of Q-LAIV by 50% did not affect the average infection attack rate and the variability (Additional file 2: Table S7). Only when full protection of Q-LAIV was assumed, childhood vaccination decreased the average infection attack rate and the variability. We also found that a simultaneous increase of the duration of protection of Q-LAIV to 2 years and the efficacy of Q-LAIV by 50% did not substantially affect the average infection attack rate and the variability (Additional file 2: Figure S4 and S5), nor the average ICER (a decrease from €3944 per QALY gained to €2476 per QALY gained (Fig. 3)).

Figure 3 shows results of the univariate sensitivity analysis (more detailed results available in Additional file 2: Table S8). We found that the childhood program remained cost-effective for a threshold of €20,000 per QALY gained when the healthcare payer’s perspective was adopted (ICER, €12,886 per QALY gained). Furthermore, the childhood program was not sensitive to the vaccine price of Q-LAIV. When the price Q-LAIV increased from €3.79 to €10.71 or further to €19.32, the average ICER increased to €7070 or €10,850 per QALY gained, respectively. In contrast, exclusion of indirect healthcare costs in life-years gained had high impact on the ICER. For this scenario, the childhood vaccination program was found to be cost-saving. Assuming a shorter life expectancy for prevented influenza deaths aged 70 years and older decreased the QALYs gain as well as the indirect healthcare costs in gained life-years, resulting in an average ICER of €700 per QALY gained.

### Number of seasons with large influenza epidemics

**Fig. 4:**
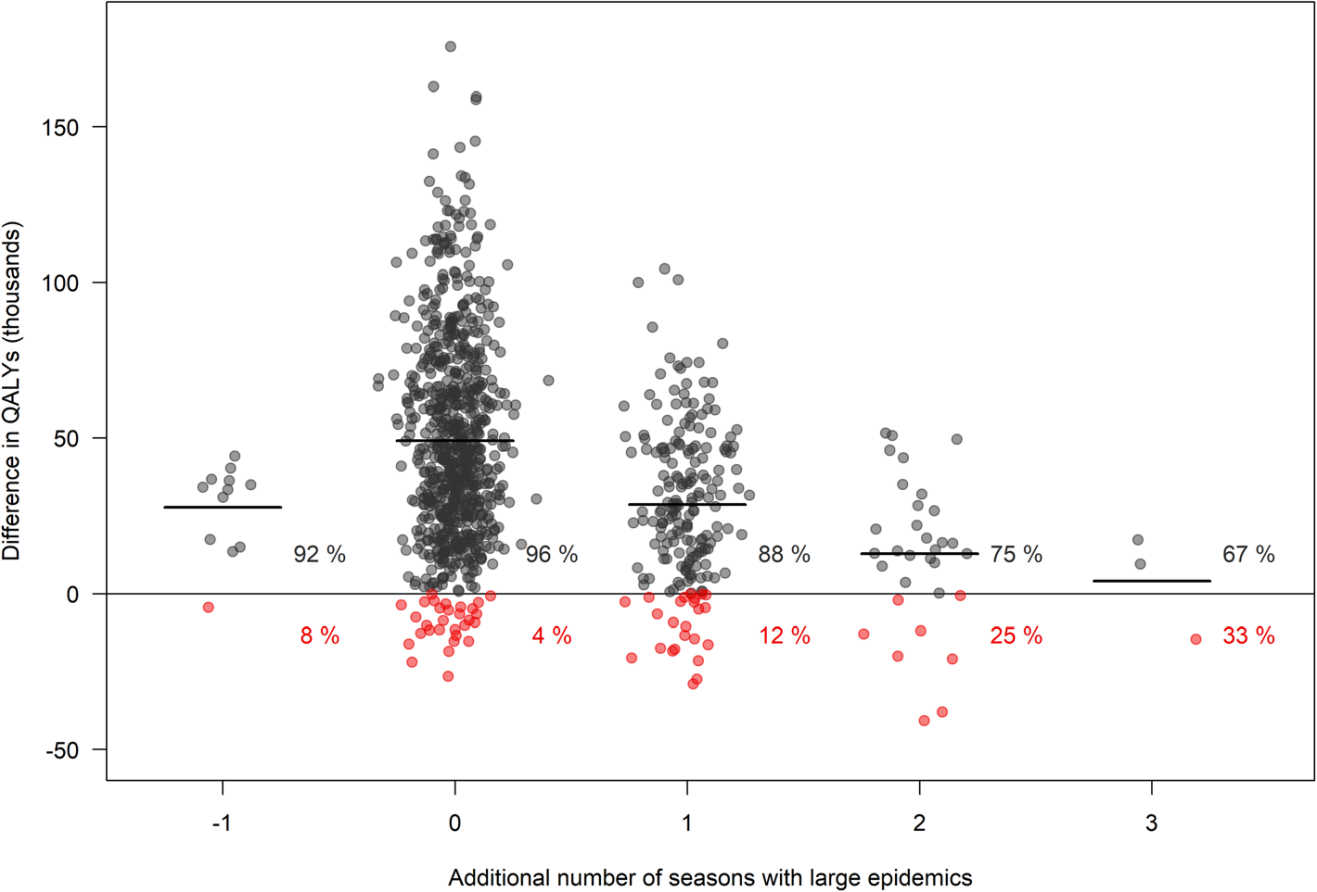
The relation between the difference in QALYs and the additional number of seasons with large influenza epidemics after extending the current program with vaccination of children aged 2–16 years at 50% coverage. Results are based on a time-horizon of 20 years. A large epidemic was defined as a season with a symptomatic attack rate larger than 5%. Outcomes are obtained from a probabilistic sensitivity analysis using 1000 simulations. Gray dots represent simulations with an overall QALY gain, while red dots represent simulations with an overall QALY loss. The black line indicates the average QALY difference. Gray and red percentages represent the proportion of simulations with an overall QALY gain and QALY loss out of the total number of simulations with that specific increase of number of seasons with large epidemics

Figure 4 shows the impact of childhood vaccination on the number of seasons with large epidemics (defined as a symptomatic attack rate larger than 5%). In 23.3% of the simulations, childhood vaccination increased the number of seasons with large epidemics as compared to the current program over a period of 20 years. In 1.2% of the simulations, the childhood vaccination program decreased the number of seasons with large epidemics. The simulations with a higher number of seasons with large epidemics tended to have a lower QALY gain and a higher probability to have a net health loss.

### Probability that childhood influenza vaccination results in a net health loss

The probabilistic sensitivity analysis demonstrated that the vaccination of children aged 2–16 years led in 6.4% of the simulations to a net QALY loss. Simulations with a net QALY loss showed relatively more often an increase of the number of seasons with large epidemics (Fig. 4). For instance, 4% of the simulations without an increase of the number of seasons with large epidemics resulted in a net loss of QALYs, while this proportion was 12% for simulations predicting one additional season with a large epidemic and 25% for simulations predicting two additional seasons with large epidemics.

## Discussion

### Interpretation of the main findings

This modeling study indicates that the introduction of an influenza vaccination program for children in the Netherlands is cost-effective for the conventional Dutch threshold of €20,000 per QALY. However, the program is not estimated to be cost-effective when only outcomes among children themselves are considered. Uncertainty analysis reveals that these average outcomes hide a wide range of outcomes. In approximately a quarter of the simulations, the introduction of a childhood vaccination program results in an increase in seasons with a symptomatic influenza attack rate larger than 5%, which is expected to cause serious strain on the health care system. Furthermore, 6% of the simulations results in a net health loss over 20 seasons.

We found that simulations with a net health loss were associated with an increase in seasons with large epidemics. This suggests that the additional burden in seasons with larger epidemics offsets the gain of health in seasons with smaller epidemics. Uncertainty analysis suggests that the size and frequency of these large epidemics are affected by the values of epidemiologic parameters, such as the reproduction number and the waning rate of immunity acquired through natural infection. Future studies that provide more precise estimates of these epidemiologic parameter values would help to further increase the precision of the frequency and risk of large epidemics, and the risk of a net health loss.

### Strengths and limitations

This cost-effectiveness analysis relies on an influenza transmission model that captures the long-term infection dynamics as well as seasonal variability in vaccine effectiveness and epidemic size. Accounting for both these aspects is essential to estimate the long-term consequences of influenza vaccination programs [43]. The transmission model has been calibrated to the reported number of GP visits with influenza-like illness in the Netherlands over 11 seasons (2003/2004 to 2014/2015). The outcome was subjected to an extensive sensitivity analysis, which allowed for detection of any undesirable effects of the childhood vaccination program.

Throughout this study, we measured the duration of protection against the circulating influenza strains, rather than protection against specific types (influenza A and B) and subtypes (A/H1N1 and A/H3N2). This allows for a direct comparison between simulation results and observations, as clinical trials and observational studies also measure protection against circulating influenza strains. This approach has been used in modeling studies where transmission models were fitted to the observed long-term dynamics [44–47]. The effect of including different influenza types and subtypes is straightforward when assuming independence between them. In Additional file 3, we show simulation results where we calibrate the model to only observational data of A/H3N2. This results in a longer duration of protection as compared to the analysis that used all influenza types and subtypes. The resulting dynamic patterns are similar to the analysis that used all influenza types and subtypes: introducing childhood influenza vaccination leads to an infection attack rate that is on average lower, but the number of seasons with large epidemics is higher. Therefore, we do not expect the specific modeling choices with respect to the multiple influenza types, subtypes, and strains to affect our conclusions.

We assumed that there is no significant evidence for one type of vaccine being more effective than another. Recently, the US Advisory Committee on Immunization Practices temporarily withdrew their recommendation for the use of Q-LAIV due to a lack of vaccine effectiveness, particularly against A/H1N1pdm09-like virus, while the inactivated influenza vaccine was effective [48, 49]. In the UK and Finland, statistically significant evidence for the effectiveness of Q-LAIV was found, and both countries continue to recommend the use of it in their national immunization programs [24]. Furthermore, one could argue that the efficacy of quadrivalent live attenuated vaccines (Q-LAIV) should be larger than trivalent inactivated vaccine (TIV) as it includes an additional B-strain. However, there are no studies that directly compared the efficacy between quadrivalent and trivalent influenza vaccines.

We took the duration of protection of influenza vaccination as one season, while some evidence suggests that LAIV may provide longer protection [28]. However, our sensitivity analysis demonstrated that a longer duration of protection of Q-LAIV would slightly increase the average impact of the vaccination program, but not mitigate the increased variability in epidemic size. Similar results were obtained for the model that focused on influenza A/H3N2 (Additional file 3). A longer duration of protection by Q-LAIV does not affect the increase in variability in epidemic size because vaccinated children will also be vaccinated in the next season (which seems most common in practice); only children that leave the program at 17 years of age benefit from a longer duration of vaccine protection. Only when vaccination provides full protection, childhood influenza vaccination is expected to reduce the variability in epidemic size. Therefore, the development of improved influenza vaccines that have consistent efficacy over consecutive seasons seems necessary.

Several recent studies suggest that the first influenza infections in early childhood determine the immune response against subsequent infections [50, 51]. If repetitive vaccination in early childhood would interfere with imprinting [52], our analysis may overestimate the impact of childhood influenza vaccination; if repetitive vaccination would promote imprinting, our analysis may underestimate the impact of influenza vaccination. Moreover, adapting a lifetime time-horizon would be required in order to estimate the full impact of influenza vaccination.

We used the tendered Dutch price of TIV as vaccine cost of Q-LAIV, while in other countries Q-LAIV is relatively more expensive than TIV [53]. Moreover, vaccination costs could also be higher, since costs of adverse events of vaccination and implementation costs of the program were not included. However, a sensitivity analysis on the vaccine price showed that the cost-effectiveness of childhood vaccination program was not sensitive to vaccination costs.

Finally, we did not consider some possible long-term effects of influenza infection, such as the acute respiratory distress syndrome and a rapid onset of inflammation in the lungs that may cause lifelong disability. However, this complication is rare, and exclusion of it reflects a conservative approach.

### Comparison to other studies

Our finding that childhood influenza vaccination is on average cost-effective on the population level is consistent with previous dynamic modeling studies in England and Wales and in Germany that reported a childhood vaccination program to be cost-effective to their national willingness-to-pay thresholds [8, 54–56]. Our finding that childhood influenza vaccination could lead to more seasons with large epidemics, and that there is a possibility of a net health loss, has not been reported before. These undesirable outcomes only arise when the analysis accounts for the long-term infection dynamics in the population and for the seasonal variation in vaccine effectiveness and epidemic size [43]. Earlier analyses accounted for either the long-term infection dynamics [55, 56], or the seasonal variability in vaccine effectiveness and epidemic size [8, 54], but not both. Therefore, they could not have detected the potential existence of such undesirable effects.

The finding that a childhood influenza vaccination program is expected to be not cost-effective for the target-group itself differs from a previous dynamic modeling study for England and Wales [8]. Using a “type and subtype”-specific model, the study for England and Wales estimated the benefits of the childhood program for 14 seasons without linkage of the seasons; hence, the study focuses on the impact and seasonal variability of childhood vaccination in the first season after implementation of the program, but does not account for the gains and losses of immunity due to natural infection and vaccination over time. We modeled immunity against the circulating strains, focusing on the impact of the childhood vaccination over 20 linked seasons while capturing the gains and losses of immunity over time.

### Implications for policy makers

Results of this study are of direct interest to public health policy makers. We expect that a childhood influenza vaccination program prevents significant burden and is on average cost-effective for the entire population.

A policy decision on the introduction of a childhood influenza vaccination program requires more than a single cost-effectiveness analysis such as the consideration of the acceptability of vaccination [57]. Most of the burden prevented by childhood vaccination is not among those vaccinated but among older adults through indirect protection, and we found that childhood vaccination is not cost-effective for the target-group itself. As a non-uniform distribution of benefits may raise issues on the fairness and acceptability of the vaccination program [57, 58], policy makers could also consider whether the burden of influenza among children themselves justifies the healthcare use and costs of a vaccination program. Furthermore, as young children are not in a position of decision-making, a childhood vaccination program should also consider the support among parents of the children; a recent Internet survey among Dutch parents measured the intention to vaccinate children against influenza at 15% [59].

The finding that childhood influenza vaccination increases the frequency of seasons with a symptomatic attack rate larger than 5% in approximately a quarter of the simulations should be a source of concern. This threshold of 5% was based on the Netherlands 2017/2018 season, in which hospitals reported to have difficulties managing bed and staff capacity due to a high number of elderly cases requiring hospitalization combined with increased sick leave among qualified staff [42]. A symptomatic attack rate of 5% is 2.8 (5/1.78) times higher than the average symptomatic attack rate we estimated for the current program, and the use of a lower threshold would increase the proportion of simulation in which childhood influenza vaccination leads to an increased frequency in seasons with large epidemics. To deal with more frequent and higher peaks in healthcare demand during influenza epidemics, hospitals would need to increase their capacity. Furthermore, an increased strain on the healthcare system may cause substantial outbreak costs, such as impaired treatment outcomes or canceled routine surgeries. Factoring these costs into our analysis would decrease the likelihood of childhood influenza vaccination being cost-effective.

Our finding of a net health loss upon the introduction of a childhood vaccination program in 6% of the simulations also needs to be taken into account in policy making. However, there are no clear guidelines how this risk should be weighed. Most preventive measures that are considered in cost-effectiveness analyses have a risk of a net health loss close to 0%; hence, a 6% probability of a net health loss for the entire vaccination program in this modeling study is remarkable. A well-known example of a preventive measure with a high risk of health loss is the introduction of a rubella vaccination program: at intermediate vaccination coverage, the population risk of congenital rubella syndrome is higher than it would be without a vaccination program [60]. This has been dubbed a “perverse outcome of mass vaccination” [61]. In the past, the Netherlands has been very careful in avoiding such a perverse outcome [62].

### Implications for monitoring influenza after introducing childhood vaccination

The counterintuitive finding that childhood influenza vaccination increases the number of seasons with a symptomatic attack rate larger than 5% in approximately a quarter of the simulations warrants proper monitoring of influenza immunity and infections to accompany any childhood influenza vaccination program. It also suggests there is merit in future analyses of available observations in populations with substantial influenza vaccine coverage in children over multiple years. As the roll-out of the childhood influenza vaccination programs in the UK has not been completed yet, it is currently too soon to see any such effects. The USA, where the influenza vaccination coverage in children has been in the range of 50 to 60% over the last decade, might provide an interesting population for such analyses. Although not necessarily explained by the implementation of childhood influenza vaccination, we observed that the USA encountered in 2017/2018 the largest seasonal influenza epidemic across all age groups since 2003/2004 [63, 64]. A proper analysis requires regions that differ in vaccination coverage for children but are otherwise comparable.

## Conclusions

This modeling study indicates that a childhood influenza vaccination program in the Netherlands is expected to be on average cost-effective. The childhood vaccination program is not expected to be cost-effective when only outcomes among children themselves are included. In approximately a quarter of the simulations, introducing a childhood influenza vaccination program increases the frequency of seasons with a symptomatic attack rate larger than 5%, which is expected to cause serious strain on the health care system. The possibility of a net loss of health cannot be excluded.

## Supplementary information

**Additional file 1.** Supplemental methods.

**Additional file 2.** Supplemental results.

**Additional file 3.** Supplemental simulations on A/H3N2.

## Data Availability

All relevant data are within the paper. Further details are available from the first author on reasonable request.

## Abbreviations

GP: General practitioner
ICER: Incremental cost-effectiveness ratio
LAIV: Live-attenuated influenza vaccine
QALY: Quality-adjusted life-year
Q-LAIV: Quadrivalent live-attenuated influenza vaccine
TIV: Trivalent inactivated vaccine
