# Supplementary material for: Vaccinating children against influenza: overall cost-effective with potential for undesirable outcomes
Source: BMC Med. 2020 Jan 14;18:11. doi: 10.1186/s12916-019-1471-x (PMC6958762; doi:10.1186/s12916-019-1471-x)
Supplement: Supplementary file 1 — Additional file 1. Supplemental methods. [file 12916_2019_1471_MOESM1_ESM.docx]

**Additional file 1**

Supplemental Methods to:

*Title:* Vaccinating children against influenza: overall cost-effective with potential for undesirable outcomes

Pieter. T. de Boer, Jantien A. Backer, Albert Jan van Hoek, Jacco Wallinga

Centre for Infectious Disease Control, National Institute for Public Health and the Environment, Bilthoven, The Netherlands

## Description and input parameters of dynamic transmission model

The dynamic transmission model was developed previously and fitted to influenza attack rates from the Netherlands and vaccine effectiveness values from the literature [1]. A structured compartmental model (Figure S1) is used to calculate the seasonal infection attack rate in each age group and risk group. The influenza epidemics end due to depletion of susceptibles rather than seasonal forcing, which allows for the use of final size equations to approximate the epidemic size. Each time step of one influenza season consists of three stages. Before the start of the season, people are vaccinated according to the vaccination coverage of their risk and age class, but whether they develop an antibody response depends on their age, captured by the vaccine take (purple arrows). During the season, the number of infections and distribution over the various groups is determined by the virus transmissibility and the contact structure between age and sex classes (red arrows). Persons that respond to vaccination can still be infected, depending on the how well the vaccine strain matches the circulating virus strain. The vaccine match can vary per season. At the end of the season, the population ages by one year and new-borns enter the population. A part of the infected population becomes susceptible to infection again due to antigenic drift, as the circulating strain less and less resembles the virus strain that caused the initial infection. This is modelled as immunity waning where individuals go from an immune state to a susceptible state (blue arrows). The waning immunity rate can also vary per season. Finally, vaccinated individuals return to an unvaccinated status as vaccine protection is assumed to last one season. We assume consistent vaccination, where individuals that are vaccinated in one season will also be vaccinated in the next season, as this best represents the current practice.


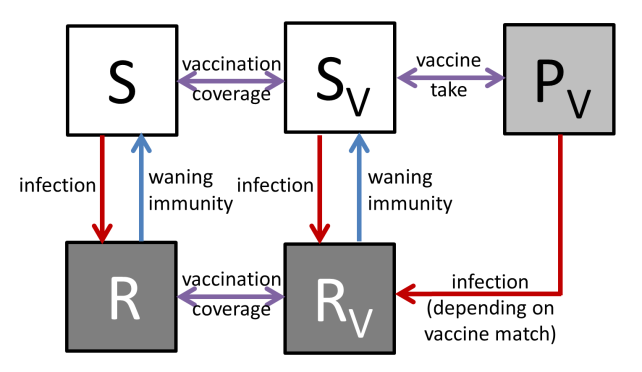


Figure S1: Overview of the influenza model. The model is a compartmental model for discrete time steps of one year, with classification according to vaccination and infection status: fully susceptible (S and S_V_), immune through natural infection (R and R_V_), and partially protected by vaccination (P_V_), where V denotes a vaccinated compartment. The model lacks an explicit infectious compartment because of the use of final size calculations.

Table S1 describes the input values for the simulation model. Model parameters were estimated based on infection attack rates of 11 seasons (2003 – 2015) in the Netherlands and on vaccine effectiveness values from literature. It was verified in the fitting procedure that the proportion of simulations with small values of the effective reproduction number and hence long seasons, is very small.

Table S1: Model parameters used for simulations. Distributions are estimated for the natural immunity duration and vaccine match, (mean and 95% credible intervals in table) as they vary by season.

| Parameter | Value | Notes |
| --- | --- | --- |
| Number of age classes | 100 | From 0 to 99+ years of age |
| Number of risk classes | 2 | High risk group consists of persons with high risk of complications and are all invited for vaccination |
| Default population demography | 2015 | Statistics Netherlands; simulations use projections up to 2045 |
| Contact matrix | - | 200x200 matrix with contact rates between each age class and sex [2] |
| Vaccination coverage in current programme | 21% | Low risk group under 60 years of age is unvaccinated , low risk group above 60 years of age are invited for vaccination; coverage in both risk groups increases with age [3]. The overall coverage is 21%, varying from 2.9% in <20 year-olds, 8.7% in 20-59 year-olds and 65.9% in ≥60 year-olds. |
| Vaccine take | 100% (children) to 30% (99+) | Percentage of vaccinated persons who develop an immune response upon vaccination, depending on age, estimated from seroconversion rates [1] |
| Basic reproduction number | 1.8 (1.3 – 2.7) | Estimated, see Backer 2019 [1] |
| Mean duration natural immunity (95% CI) | 5.1 (2.9 – 8.2) years | Estimated, see Backer 2019 [1] |
| Sd duration natural immunity (95% CI) | 0.91 (0.27 – 1.9) years | Estimated, see Backer 2019 [1] |
| Duration vaccine protection TIV | 1 year | Assumption |
| Mean vaccine match (95% CI) | 0.56 (0.49 – 0.66) | Estimated, see Backer 2019 [1] |
| Sd vaccine match (95% CI) | 0.11 (0.078 – 0.14) | Estimated, see Backer 2019 [1] |

## Outcome probabilities

The number of infections of the dynamic transmission model by age and risk-group were converted to symptomatic infections, GP visits, hospitalizations and deaths using outcome probabilities from the literature (see more details in [1]). The resulting symptom probability after infection is 0.30 (0.27 - 0.34) [4]. The probability of visiting a GP given symptomatic influenza was estimated using data from an online survey among influenza-like-illness patients [5]. Probability distributions of hospitalisation and mortality given infection, per risk and age group, were based on influenza-associated respiratory death rates from respiratory diagnoses for age groups under 60 years of age [6], and all-cause influenza-associated death rates over [7] 60 years of age, the fraction of influenza deaths occurring in the hospital [8], the mortality rate per hospitalisation [6], and the infection attack rate (inferred in the dynamic transmission model) and are shown in Table S2.

Table S2: Mean and standard deviation of probability of hospitalization and death given infection by age group and risk group. Please note, infection includes symptomatic as well as asymptomatic infections)

| Age group (years) | Probability of hospitalization given infection (×10^3^) | | | | Probability of mortality given infection (×10^3^) | | | |
| --- | --- | --- | --- | --- | --- | --- | --- | --- |
|  | Low risk | | High risk | | Low risk | | High risk | |
| 0–4 | 21 | (1.4) | 21 | (1.3) | 0.009 | (0.00059) | 0.36 | (0.021) |
| 5–14 | 0.87 | (0.067) | 5.6 | (0.3) | 0.00037 | (0.000029) | 0.096 | (0.005) |
| 15–44 | 1.1 | (0.039) | 6.4 | (0.26) | 0.007 | (0.00024) | 0.26 | (0.01) |
| 45–59 | 1.7 | (0.051) | 11 | (0.46) | 0.014 | (0.00042) | 0.56 | (0.024) |
| 60–64 | 3 | (0.099) | 9.5 | (0.37) | 0.084 | (0.0037) | 2.8 | (0.12) |
| 65–69 | 4.3 | (0.15) | 8.4 | (0.29) | 1.2 | (0.042) | 5.5 | (0.19) |
| 70–74 | 8 | (0.18) | 16 | (0.35) | 2.3 | (0.05) | 10 | (0.23) |
| 75–79 | 14 | (0.5) | 27 | (0.99) | 5.1 | (0.19) | 23 | (0.86) |
| 80–84 | 38 | (1.3) | 75 | (2.5) | 14 | (0.48) | 64 | (2.2) |
| 85–99 | 93 | (2) | 180 | (3.8) | 35 | (0.74) | 160 | (3.3) |

## Economic input parameters

Clinical outcomes were converted to quality-adjusted life years (QALYs) lost and costs by multiplying the number of symptomatic infection, GP visits, hospitalizations and deaths with the associated burden estimates. These are shown in Table S3 and described in more detail below.

### QALY losses

QALY losses of medically attended influenza and hospitalized influenza are based on adults with a lower-respiratory tract infection and community acquired-pneumonia, respectively [9, 10]. These QALY losses were estimated using the EuroQol 5-dimension (EQ-5D) instrument and Dutch EQ-5D tariffs. The QALY loss of a non-medically attended case was calculated by multiplying the QALY loss of a medically attended influenza case with the ratio of the QALY loss of non-medically attended and medically attended influenza-like-illness patients from a study in Belgium [11]. In the sensitivity analysis, we explored previously published influenza-related QALY losses as presented by Lugner et al. [12].

### Costs

Direct healthcare cost of a GP visit for children was obtained from a Dutch study among influenza-like illness cases aged <5 years [13]. This study used a web-based survey to compare the resource use and costs of children that are attending day care versus children that are not attending day care. To estimate the average costs per GP visit, we first divided the average costs per child attributed to GP visits or laboratory use by the proportion of children requiring these types of care for these groups separately. For the average costs per child attributed to medication use we assumed that 52% of these costs were related to prescribed drugs [14]. Then, we added up these costs, and calculated a weighted average by using the assumption that 47% of children in the Netherlands attend day care [15]. In absence of cost data for children above the age of 5 years, we used this estimation for all children aged 0-17 years. For influenza-related GP visits of individuals aged 18 years and older, we used published cost data of Dutch patients attending a GP due to a lower-respiratory infection [10]. Age-specific costs of an influenza-related hospitalization were based on the length of stay and proportion of days that were spend on the ICU from patients with community acquired pneumonia [16]. These lengths of stay were then multiplied with the cost per bed day on a paediatric ward (children below 18 years of age), general ward (adults 18 years of age or older) and ICU ward (regardless of age) [17]. We attributed no additional direct healthcare costs to influenza-associated deaths upon a possible prior GP visit or a hospital visit.

Indirect healthcare costs (healthcare costs unrelated to influenza in life years gained) were estimated using the life-expectancy at the age that death was averted and age-specific healthcare costs from a specifically developed tool labelled Practical Application to Include Disease Costs (PAID) [18]. This tool distinguishes healthcare costs incurred in the last year of life and costs incurred in other years by sex, age and health care provider. To avoid a possible double count of influenza-related costs, we excluded healthcare costs of the disease category ‘pneumonia and influenza’. We included costs of all healthcare providers available in the tool and the weighted average of men and women was estimated using age-specific sex distributions of the Dutch population in 2017 [19]. The total indirect healthcare costs in the remaining life years was estimated using lifetables, attributing the cost incurred in a final life year to a person that died in the lifetable and cost incurred in other years to a person that survives in the lifetable.

Patient costs of cases without medical attention included costs of over-the-counter medication. We based the proportion of patients that used over-the-counter (OTC) medication by drug class on the resource use of influenza-like-illness patients from a study in Belgium [11]. For each drug class we selected a proxy drug that we considered as the most likely drug prescribed and estimated the costs of the drugs by using standard treatment guidelines on dose and duration of treatment. For influenza patients attending a GP, we used published costs of adults that visited the GP due to a lower-respiratory infection [10]. These costs consisted of OTC medication and travel costs. For hospitalized cases, we based the patient costs on the declared costs of patients with community-acquired pneumonia-related admission. These costs consisted of travel cost to the hospital and possible paid household help, but not home-care [20].

Productivity losses due to work absence of non-hospitalized cases were estimated using data from a prospective web-based surveillance study that monitored the incidence of influenza-like-illnesses (ILIs) on the basis of self-reporting (see Friesema et al.[5] for more details on data collection and ILI definition). We used data from five consecutive winter seasons in the period 2003/2004 to 2007/2008 to estimate the proportion of employed cases that reported missed work and, if so, the number of days missed. We stratified the data by age group (15-24 years, 25-44 years, 45-59 years and ≥60 years) and by patients consulting a GP or not. Then, we converted the number of work days lost to number of working hours lost using data on average working hours per week [21], and subsequently to productivity losses using the standard tariff per missed working hour from the Dutch cost-effectiveness guideline [17]. As the estimated work absences were for the working population, we adjusted our estimates using age-specific labour participation rates [22]. For hospitalized cases, we assumed that the duration of work absence equals the length of stay plus the work absence of an ILI case with a GP visit. We estimated the number of work days lost due to influenza-related death using the friction cost method [23]. This method assumes that work absence is limited to a certain friction period, as an unemployed person has replaced the deceased person after this period. We used a friction period of 85 days [17]. In the sensitivity analysis we explored a scenario in which we estimated the number of work days lost according to the human capital approach, which considers all work days lost until the age of retirement. The human capital cost of a deceased person was estimated using life table methodology, taking into account premature death due to other causes. The productivity loss of caregivers of children with non-hospitalized influenza was estimated using the same data and methodology that was used to estimate the direct healthcare costs of GP visits of children [13]. The work absence was converted to productivity losses by using labour participation rates and average number of working hours per week of the people aged 25-44. For hospitalized children, the work absence was based on children aged <2 years with a hospitalized RSV episode [24]. In absence of data for older children, we used productivity losses of caregivers for all children aged up to 15 years.

### Sensitivity analysis of economic parameters

Economic input parameters that report a standard error and distribution were simultaneously varied in the probabilistic sensitivity analysis. In a univariate sensitivity analysis, the vaccine price of Q-LAIV was increased to €10.71 (list price of TIV [25]) and €19.32 (Q-LAIV price of the US Centers for Disease Control and prevention [26], converted to euros using purchasing power parities [27]). We also conducted a scenario from the healthcare payer’s perspective (excluding direct non-healthcare costs and productivity losses) and a scenario that excluded indirect healthcare costs in gained life years. For measuring productivity losses of premature influenza-associated deaths we performed a scenario in which the human capital approach was used, valuing all productivity losses until the age of retirement. With regard to health effects, we explored QALY losses of influenza illness as previously used by Lugner et al.[12] and performed a scenario in which the life-expectancies of premature influenza-associated deaths above the age of 70 years were halved. The latter scenario assumes that elderly dying because of influenza may be less healthy than the general population.

Table S3: Economic inputs of the analysis. In the probabilistic sensitivity analysis, the parameter inputs were simultaneously varied using the standard error and distribution shown. In the univariate sensitivity analysis, the parameter inputs were varied one-by-one.

| Parameter | Deterministic | Standard error | Distribution for PSA | Univariate SA | Reference / notes |
| --- | --- | --- | --- | --- | --- |
| *Vaccination costs* |  |  |  |  |  |
| Q-LAIV per dose | €3.59 |  |  | €10.71; €19.32 | Tendered TIV price in the Netherlands [28]; In the univariate SA we used the list price of TIV [25]and the Q-LAIV price of the US CDC [26]. |
| Administration per dose | €11.36 |  |  |  | SNPG, 2017 [29] |
| *Direct healthcare costs* |  |  |  |  |  |
| GP visit (€) |  |  |  |  |  |
| 0-17y | €52.20 | 1.54 | Normal |  | Enserink, 2014 [13] |
| ≥18y | €82.86 | 2.69 | Normal |  | Mangen, 2015 [10] |
| Hospitalization | LOS / % of LOS on ICU |  |  |  | Rozenbaum, 2015 [16] |
| 0-9y | 3.60/0.42% | 0.03 / - | Normal |  |  |
| 10-17y | 4.56/1.60% | 0.16 / - | Normal |  |  |
| 18-24y | 5.18/7.38% | 0.05 / - | Normal |  |  |
| 25-44y | 6.87/9.63% | 0.06 / - | Normal |  |  |
| 45-59y | 7.72/7.95% | 0.06 / - | Normal |  |  |
| 60-74y | 8.02/5.34% | 0.05 / - | Normal |  |  |
| ≥75y | 7.78/1.47% | 0.06 / - | Normal |  |  |
| Cost per bed day |  |  |  |  | Dutch CE guideline, 2016 [17] |
| Paediatric ward | €642 |  |  |  |  |
| General ward | €487 |  |  |  |  |
| ICU | €2,062 |  |  |  |  |
| *Indirect healthcare costs* | Age-specific |  |  | Excluded | Statistics Netherlands, 2017; Van Baal, 2011 [18] |
| *Direct non-healthcare costs* |  |  |  |  |  |
| Non-medically attended | €7.04 | 0.16 | Normal |  | Bilcke, 2014 [11] drug costs |
| GP visit | €21.45 | 1.97 | Normal |  | Mangen, 2015 [10] |
| Hospitalization | €128.21 | 25.64 | Normal |  | Van Werkhoven, 2017 [20] |
| *Productivity losses* |  |  |  |  |  |
| Employed patient (days of absence) |  |  |  |  |  |
| Non-medically attended |  |  |  |  | Self-reported data from a web-based surveillance system monitoring influenza symptoms (see [5] for more details) |
| 15-24 | 3.29 | 0.15 | Normal |  |  |
| 25-44 | 3.41 | 0.04 | Normal |  |  |
| 45-59 | 3.89 | 0.06 | Normal |  |  |
| 60-69 | 3.49 | 0.17 | Normal |  |  |
| GP visit |  |  |  |  |  |
| 15-24 | 5.19 | 0.30 | Normal |  |  |
| 25-44 | 6.20 | 0.12 | Normal |  |  |
| 45-59 | 6.88 | 0.16 | Normal |  |  |
| 60-69 | 5.88 | 0.22 | Normal |  |  |
| Hospitalization | Equal to LOS |  |  |  | Assumption |
| Death | 85 |  |  | HCA | Dutch CE guideline, 2016 [17], friction period |
| Employed caregiver (days of absence) |  |  |  |  |  |
| Non-hospitalized | 0.61 | 0.078 | Normal |  | Enserink, 2014 [13] |
| Hospitalized | 2.00 | 0.26 | Normal |  | Miedema, 2001 [24] |
| Average number of work hours per week | Age-specific |  |  |  | Statistics Netherlands [21] |
| Labour participation rate | Age-specific |  |  |  | Statistics Netherlands [22] |
| Productivity loss per hour | €35.55 |  |  |  | Dutch CE guideline, 2016 [17] |
| *QALY losses* |  |  |  |  |  |
| Non-medically attended | 0.0038 | 0.0004 | Normal | 0.0083 | Adjusted QALY loss of GP visit using Bilcke, 2014 [11] |
| GP visit | 0.0045 | 0.00051 | Normal | 0.010 | Mangen, 2015 [10] ; Univariate SA based on Lugner, 2010 [12] |
| Hospitalization | 0.0118 | 0.00030 | Normal | 0.0217 | Mangen, 2013 [9]; Univariate SA based on Lugner, 2010 [12] |
| Death |  |  |  |  |  |
| Life years lost | Age-specific |  |  | Halved for ≥70y | Statistics Netherlands [30] |
| QoL population norms | Age-specific |  |  |  | Versteegh, 2016 [31] |

CE: Cost-effectiveness, GP: General practitioner, HCA: Human capital approach, ICU: Intense care unit, LOS: Length of stay, QALY: Quality-adjusted life year, QoL: Quality of life, Q-LAIV: Quadrivalent live-attenuated influenza vaccine, PSA: Probabilistic sensitivity analysis, SA: Sensitivity analysis, SNPG: Stichting nationaal programma grieppreventie, TIV: Trivalent inactivated vaccine.

### Sensitivity analysis of duration of protection of Q-LAIV

In the main analysis, Q-LAIV used in children was assumed to have similar characteristics as TIV used in the current program, i.e. a vaccine protection of 1 year and an average vaccine match of 56%. To allow for a longer duration of protection of Q-LAIV, the original model is extended with two additional compartments (Fig. S1.1B): one compartment to hold vaccinated low-risk children (Q_V_) and one compartment to hold low-risk children that were previously vaccinated (Q). High-risk children remain vaccinated with TIV, as they could have a contra-indication to Q-LAIV. In the extended model, Q-LAIV protection lasts at least one year, after which children that are not vaccinated in the next season move to the Q compartment. When the duration of protection of Q-LAIV (D_Q_) is less than two years, they will be proportionally divided between the Q (D_Q_ – 1)and S compartment (2 – D_Q_). When the total Q-LAIV protection is more than two years the vaccine protection wanes with an annual rate of (1/(D_Q_ – 1)). The children in the Q_V_ and Q compartments can still be infected, depending on the vaccine match. In the new model, Q-LAIV can also be assumed to have a higher efficacy than TIV by multiplying the vaccine match with a factor.

| **A Original model** | **B Model with extended protection by QLAIV** |
| --- | --- |
| 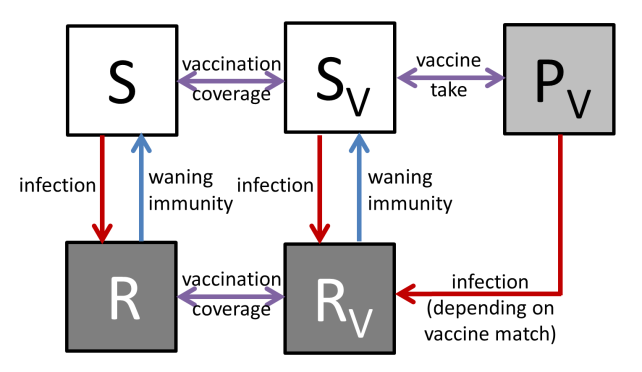 | 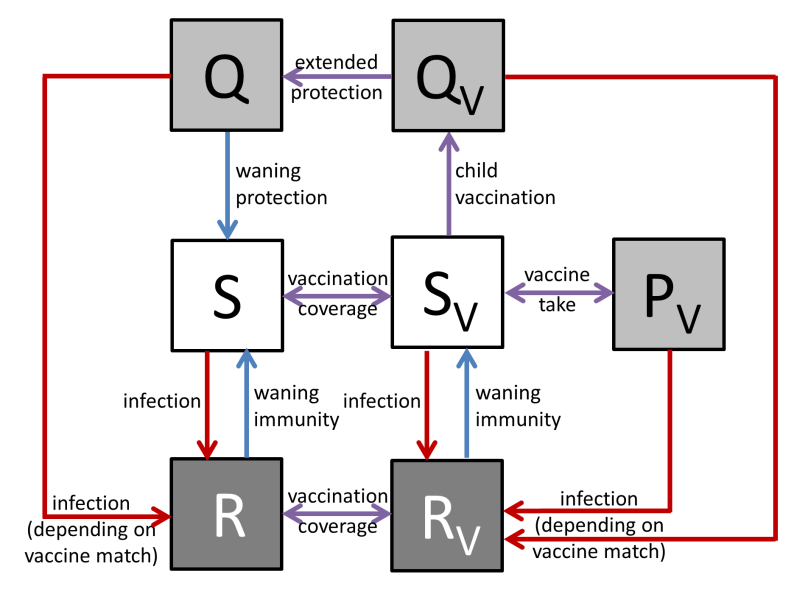 |

Figure S2: Overview of the influenza model. A) Original compartmental model for discrete time steps of one year, with classification according to vaccination and infection status: fully susceptible (S and SV), immune through natural infection (R and RV), and partially protected by vaccination (PV), where subscript V denotes a vaccinated compartment. The model lacks an explicit infectious compartment because of the use of final size calculations. B) Adapted model to allow for extended protection by QLAIV. When vaccinated low-risk children in the Q_V_ compartment are not vaccinated the following season, they move to the unvaccinated Q compartment, where they will still be partially protected against infection for an extended period until the vaccine protection wanes.

## References

1. Backer JA, Wallinga J, Meijer A, Donker GA, van der Hoek W, van Boven M. The impact of influenza vaccination on infection, hospitalisation and mortality in the Netherlands between 2003 and 2015. Epidemics 2019, 26:77-85.

2. Van de Kassteele J, Van Eijkeren J, Wallinga J. Efficient estimation of age-specific contact rates between men and women. Ann Appl Stat 2017, 11:320.

3. Tacken MA, Jansen B, Mulder J, Campbell SM, Braspenning JC. Dutch influenza vaccination rate drops for fifth consecutive year. Vaccine 2015, 33(38):4886-4891.

4. Carrat F, Vergu E, Ferguson NM, Lemaitre M, Cauchemez S, Leach S, Valleron AJ. Time lines of infection and disease in human influenza: a review of volunteer challenge studies. Am J Epidemiol 2008, 167(7):775-785.

5. Friesema IH, Koppeschaar CE, Donker GA, Dijkstra F, van Noort SP, Smallenburg R, van der Hoek W, van der Sande MA. Internet-based monitoring of influenza-like illness in the general population: experience of five influenza seasons in The Netherlands. Vaccine 2009, 27(45):6353-6357.

6. Cromer D, van Hoek AJ, Jit M, Edmunds WJ, Fleming D, Miller E. The burden of influenza in England by age and clinical risk group: a statistical analysis to inform vaccine policy. J Infect 2014, 68(4):363-371.

7. McDonald SA, van Wijhe M, van Asten L, van der Hoek W, Wallinga J. Years of life lost due to influenza-attributable mortality in older adults in the Netherlands: a competing risks approach. Am J Epidemiol 2018, 187(8):1791-1798.

8. Matias G, Taylor RJ, Haguinet F, Schuck-Paim C, Lustig RL, Fleming DM. Modelling estimates of age-specific influenza-related hospitalisation and mortality in the United Kingdom. BMC Public Health 2016, 16:481.

9. Mangen MJ, Bonten MJ, de Wit GA. Rationale and design of the costs, health status and outcomes in community-acquired pneumonia (CHO-CAP) study in elderly persons hospitalized with CAP. BMC Infect Dis 2013, 13:597.

10. Mangen MJ, Rozenbaum MH, Huijts SM, van Werkhoven CH, Postma DF, Atwood M, van Deursen AM, van der Ende A, Grobbee DE, Sanders EA *et al*. Cost-effectiveness of adult pneumococcal conjugate vaccination in the Netherlands. Eur Respir J 2015, 46(5):1407-1416.

11. Bilcke J, Coenen S, Beutels P. Influenza-like-illness and clinically diagnosed flu: disease burden, costs and quality of life for patients seeking ambulatory care or no professional care at all. PLoS One 2014, 9(7):e102634.

12. Lugner AK, Mylius SD, Wallinga J. Dynamic versus static models in cost-effectiveness analyses of anti-viral drug therapy to mitigate an influenza pandemic. Health Econ 2010, 19(5):518-531.

13. Enserink R, Lugner A, Suijkerbuijk A, Bruijning-Verhagen P, Smit HA, van Pelt W. Gastrointestinal and respiratory illness in children that do and do not attend child day care centers: a cost-of-illness study. PLoS One 2014, 9(8):e104940.

14. Dekker ARJ. Rational antibiotic prescribing for children with respiratory tract infections; Chapter 5: Cost-effectiveness analysis of a general practitioner- and parentdirected intervention to reduce antibiotic prescribing for children with respiratory tract infections in primary care (Doctoral thesis). Utrecht, the Netherlands; 2018.

15. Statistics Netherlands. Children and yearly hours spend in formal day care by type of care and age of the child (at end of the year), 2015 (in Dutch) 2016 https://www.cbs.nl/-/media/_excel/2016/23/kinderopvangtoeslag.xlsx. Accessed at Sep 1 2018.

16. Rozenbaum MH, Mangen MJ, Huijts SM, van der Werf TS, Postma MJ. Incidence, direct costs and duration of hospitalization of patients hospitalized with community acquired pneumonia: A nationwide retrospective claims database analysis. Vaccine 2015, 33(28):3193-3199.

17. National Health Care Institute. Guideline for economic evaluations in healthcare 2016 https://english.zorginstituutnederland.nl/publications/reports/2016/06/16/guideline-for-economic-evaluations-in-healthcare. Accessed at Dec 1 2017.

18. van Baal PH, Wong A, Slobbe LC, Polder JJ, Brouwer WB, de Wit GA. Standardizing the inclusion of indirect medical costs in economic evaluations. Pharmacoeconomics 2011, 29(3):175-187.

19. Statistics Netherlands. Population; Gender, age, marital status and region, 1 January 2017 http://statline.cbs.nl/Statweb/publication/?DM=SLNL&PA=03759ned&D1=0-2&D2=0-117&D3=0&D4=l&HDR=T&STB=G2,G3,G1&VW=T. Accessed at Dec 1 2017.

20. van Werkhoven CH, Postma DF, Mangen MJ, Oosterheert JJ, Bonten MJ, group C-Ss. Cost-effectiveness of antibiotic treatment strategies for community-acquired pneumonia: results from a cluster randomized cross-over trial. BMC Infect Dis 2017, 17(1):52.

21. Statistics Netherlands. Working population; working time [In Dutch] 2017 https://statline.cbs.nl/Statweb/publication/?DM=SLNL&PA=82647ned&D1=a&D2=0&D3=1-8&D4=74&HDR=G3&STB=G1,G2,T&P=T&VW=T. Accessed at 1 Feb 2019.

22. Statistics Netherlands. Labour participation; Key figures 2016 http://statline.cbs.nl/Statweb/publication/?DM=SLNL&PA=82309NED&D1=22-23&D2=a&D3=18-22&D4=0&D5=69&HDR=G1,T&STB=G2,G3,G4&VW=T. Accessed at 1 Dec 2017.

23. Koopmanschap MA, Rutten FF, van Ineveld BM, van Roijen L. The friction cost method for measuring indirect costs of disease. J Health Econ 1995, 14(2):171-189.

24. Miedema CJ, Kors AW, Tjon ATWE, Kimpen JL. Medical consumption and socioeconomic effects of infection with respiratory syncytial virus in The Netherlands. Pediatr Infect Dis J 2001, 20(2):160-163.

25. National Health Care Institute. Medicijnkosten.nl 2018 www.medicijnkosten.nl. Accessed at 1 Aug 2018.

26. Centers for Disease Control and Prevention (CDC). CDC Vaccine Price List 2019 https://www.cdc.gov/vaccines/programs/vfc/awardees/vaccine-management/price-list/index.html. Accessed at 21 Jan 2019.

27. Organisation for Economic Co-operation and Development (OECD). Purchasing power parities (PPP) 2018 https://data.oecd.org/conversion/purchasing-power-parities-ppp.htm. Accessed at 1 Feb 2019.

28. Stichting Nationaal Programma Grieppreventie (SNPG). News letter SNPG November 2017 for health-care organizations (In Dutch) 2017 https://www.snpg.nl/2017/11/23/nieuwsbrief-snpg-november-2017-zorgorganisaties/. Accessed at 1 Aug 2018.

29. Stichting Nationaal Programma Grieppreventie (SNPG). Fee (In Dutch) 2017 https://www.snpg.nl/article/declareren-griepvaccinaties/vergoeding/. Accessed at 1 Dec 2017.

30. Statistics Netherlands. Projected Demograpich Development, 2015-2060 [In Dutch] 2014 https://statline.cbs.nl/Statweb/publication/?DM=SLNL&PA=83224NED&D1=a&D2=0,5,10,15,20,25,30,35,40,l&VW=T. Accessed at 1 Jul 2016.

31. Versteegh MM, Vermeulen KM, Evers SMAA, de Wit GA, Prenger R, Stolk EA. Dutch Tariff for the Five-Level Version of EQ-5D. Value Health 2016, 19(4):343-352.
