# Supplementary material for: Vaccinating children against influenza: overall cost-effective with potential for undesirable outcomes
Source: BMC Med. 2020 Jan 14;18:11. doi: 10.1186/s12916-019-1471-x (PMC6958762; doi:10.1186/s12916-019-1471-x)
Supplement: Supplementary file 2 — Additional file 2. Supplemental results. [file 12916_2019_1471_MOESM2_ESM.docx]

**Additional file 2**

Supplemental results to:

*Title:* Vaccinating children against influenza: overall cost-effective with potential for undesirable outcomes

Pieter. T. de Boer, Jantien A. Backer, Albert Jan van Hoek, Jacco Wallinga

Centre for Infectious Disease Control, National Institute for Public Health and the Environment, Bilthoven, The Netherlands

A)


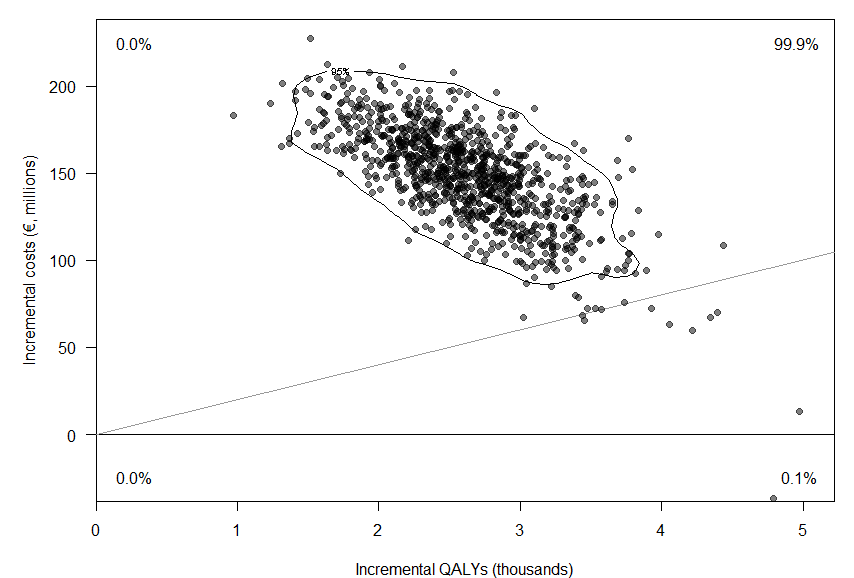


B)


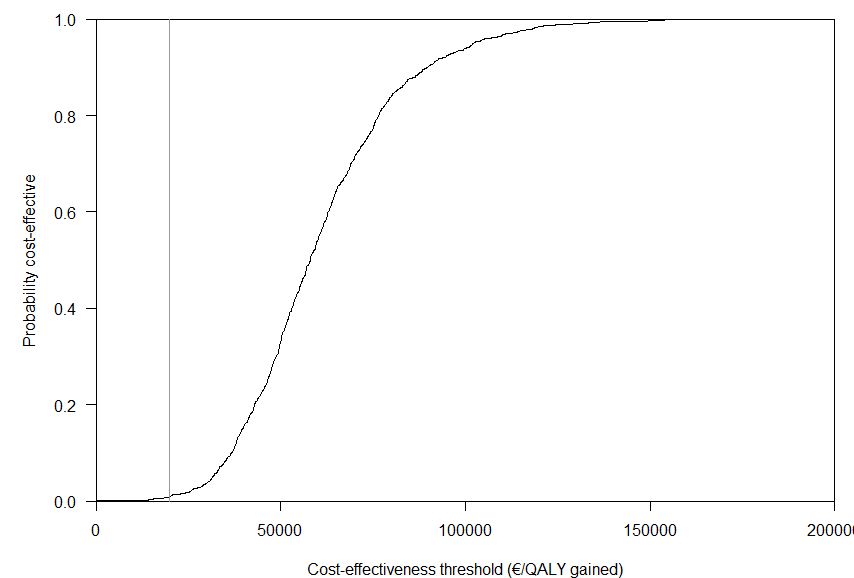


Figure S1: Probabilistic sensitivity analysis using 1,000 simulations of extending the existing program with vaccination of children aged 2-16 years at 50% coverage in the Netherlands over 20 seasons, when considering only outcomes in children aged 2-16 years. (A) The cost-effectiveness plane depicts the incremental costs and QALYs of each individual simulation. The contour line represents the 95% interval of the simulations. The grey line indicates the conventional Dutch cost-effectiveness threshold of €20,000 per QALY gained. (B) The cost-effectiveness acceptability curve depicts the proportion of cost-effective simulations over a range of cost-effectiveness thresholds. The grey line indicates the conventional Dutch cost-effectiveness threshold of €20,000 per QALY gained.


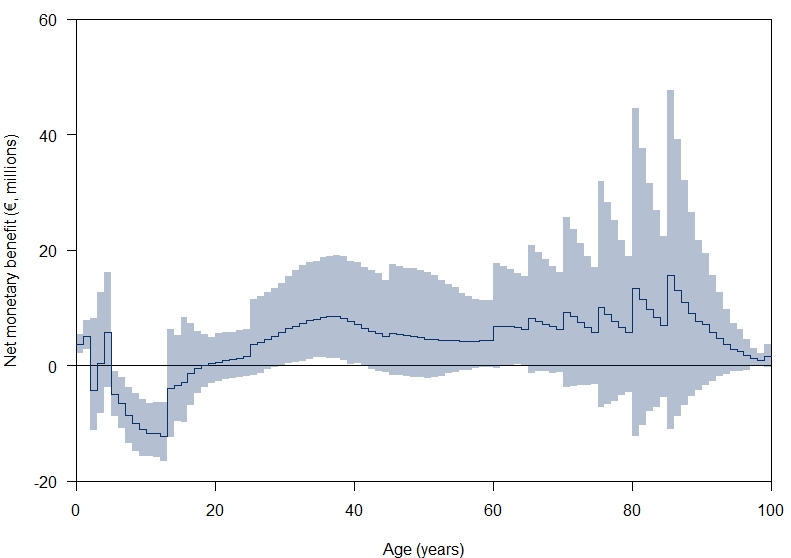


Figure S2: Net monetary benefit (NMB) of extending the current program with vaccination of children aged 2-16 at 50% coverage per yearly age group. The contours indicate the 95% confidence limits. NMB was calculated as: NMB = ΔQALYs * λ – ΔCosts, in which λ is the cost-effectiveness threshold. λ was set at €20,000 per QALY gained, the conventional Dutch cost-effectiveness threshold for preventive interventions. A positive NMB implies that the intervention is cost-effective to λ and a negative NMB implies that the simulation is not cost-effective. The saw-tooth pattern is explained by differences in outcome probabilities for hospitalization and mortality per 5-year age group, while the incidence of influenza infection is simulated per single-year age group.

Table S1: Sensitivity analysis of the targeted age-group on the clinical outcomes. The table shows the predicted 20-year annual average number of clinical events in the Netherlands in absence and presence of childhood influenza vaccination at 50% coverage. Events are shown for the entire population and for the age group 2-16 years only.

| Outcome | Symptomatic cases |  | GP visits |  | Hospitalizations |  | Deaths |  |
| --- | --- | --- | --- | --- | --- | --- | --- | --- |
|  | Mean (95% interval)^a^ | Rate^b^ | Mean (95% interval)^a^ | Rate^b^ | Mean (95% interval)^a^ | Rate^b^ | Mean (95% interval)^a^ | Rate^b^ |
| *Within the entire population* | | | |  |  |  |  |  |
| CP | 317,703 (239,785-391,519) | 1,781 | 71,552 (53,140-89,952) | 401 | 7,703 (4,960-10,391) | 43.1 | 3,234 (2,057-4,475) | 18.1 |
| CP + 2-3y | 314,455 (234,372-388,982) | 1,762 | 70,516 (51,728-88,910) | 395 | 7,477 (4,792-10,117) | 41.9 | 3,221 (2,030-4,471) | 18.0 |
| CP + 2-12y | 279,293 (163,167-367,426) | 1,565 | 62,305 (35,855-82,937) | 349 | 6,730 (3,415-9,628) | 37.7 | 3,053 (1,531-4,401) | 17.1 |
| CP + 2-16y | 260,437 (123,289-354,634) | 1,460 | 58,200 (26,929-79,538) | 326 | 6,415 (2,749-9,484) | 35.9 | 2,916 (1,208-4,353) | 16.3 |
| *Within children aged 2-16y only* | | | |  |  |  |  |  |
| CP | 86,779 (69,895-102,394) | 3,018 | 20,546 (15,968-24,684) | 714 | 1,480 (934-2,018) | 51.5 | 1.6 (.8-2.5) | 0.054 |
| CP + 2-3y | 85,015 (67,791-100,240) | 2,956 | 19,853 (15,319-23,878) | 690 | 1,295 (820-1,769) | 45.1 | 1.4 (.8-2.1) | 0.049 |
| CP + 2-12y | 66,059 (40,472-84,293) | 2,297 | 15,222 (9,134-19,692) | 529 | 932 (479-1,331) | 32.4 | 1.3 (.7-2.1) | 0.046 |
| CP + 2-16y | 55,257 (28,278-72,633) | 1,921 | 12,918 (6,428-17,287) | 449 | 865 (371-1,284) | 30.1 | 1.1 (.4-1.9) | 0.038 |

^a^: Based on 1000 simulations, 95% interval uses the 2.5% and 97.5% percentiles. ^b^: Rate per 100,000 population. CP: Current program, y: years.

Table S2: Sensitivity analysis of the coverage in healthy children. The table shows the predicted 20-year annual average number of clinical events in absence and presence of childhood influenza vaccination of children aged 2-16 years in the Netherlands over a period of 20 years. Events are shown for the entire population and for the age group 2-16 years only.

| Outcome | Symptomatic cases |  | GP visits |  | Hospitalizations |  | Deaths |  |
| --- | --- | --- | --- | --- | --- | --- | --- | --- |
|  | Mean (95% interval)^a^ | Rate^b^ | Mean (95% interval)^a^ | Rate^b^ | Mean (95% interval)^a^ | Rate^b^ | Mean (95% interval)^a^ | Rate^b^ |
| *Within the entire population* | | | |  |  |  |  |  |
| CP | 317,703 (239,785-391,519) | 1,781 | 71,552 (53,140-89,952) | 401 | 7,703 (4,960-10,391) | 43.1 | 3,234 (2,057-4,475) | 18.1 |
| CP + 2-16y at 25% coverage | 289,302 (180,230-372,265) | 1,622 | 64,885 (39,876-85,175) | 364 | 7,057 (3,836-9,998) | 39.5 | 3,087 (1,637-4,411) | 17.3 |
| CP + 2-16y at 50% coverage | 260,437 (123,289-354,634) | 1,460 | 58,200 (26,929-79,538) | 326 | 6,415 (2,749-9,484) | 35.9 | 2,916 (1,208-4,353) | 16.3 |
| CP + 2-16y at 75% coverage | 232,656 (76,510-330,295) | 1,304 | 51,817 (16,687-74,498) | 290 | 5,796 (1,682-9,016) | 32.5 | 2,727 (771-4,287) | 15.3 |
| *Within children aged 2-16y only* | | | |  |  |  |  |  |
| CP | 86,779 (69,895-102,394) | 3,018 | 20,546 (15,968-24,684) | 714 | 1,480 (934-2,018) | 51.5 | 1.6 (.8-2.5) | 0.054 |
| CP + 2-16y at 25% coverage | 70,487 (47,181-86,603) | 2,451 | 16,573 (10,683-20,896) | 576 | 1,151 (611-1,641) | 40.0 | 1.4 (.7-2.3) | 0.048 |
| CP + 2-16y at 50% coverage | 55,257 (28,278-72,633) | 1,921 | 12,918 (6,428-17,287) | 449 | 865 (371-1,284) | 30.1 | 1.1 (.4-1.9) | 0.038 |
| CP + 2-16y at 75% coverage | 41,678 (15,078-59,941) | 1,449 | 9,693 (3,509-14,182) | 337 | 628 (200-981) | 21.8 | .8 (.2-1.6) | 0.027 |

^a^: Based on 1000 simulations, 95% interval uses the 2.5% and 97.5% percentiles. ^b^: Rate per 100,000 population. CP: Current program, y: years.

Table S3: Sensitivity analysis of the targeted age-group on the clinical outcomes. The table shows the expected costs and QALY losses in absence and presence of childhood influenza vaccination at 50% coverage in the Netherlands over a period of 20 years. Outcomes are shown for the general population and for children aged 2-16 years only. Future costs and QALYs include an annual discount rate of 4% and 1.5%, respectively.

| Outcome | CP | CP + 2-3y | CP + 2-12y | CP + 2-16y |
| --- | --- | --- | --- | --- |
| ***Within the general population*** |  |  |  |  |
| *Costs (€, millions)* ^a^ |  |  |  |  |
| Vaccination | 964 | 1,004 | 1,176 | 1,250 |
| Direct HC costs | 467 | 458 | 418 | 398 |
| Indirect HC costs | - | 13 | 198 | 344 |
| Patient costs | 482 | 477 | 424 | 396 |
| Productivity loss | 2,214 | 2,198 | 2,014 | 1,911 |
| Total costs | 4,127 | 4,150 | 4,230 | 4,299 |
| *QALY loss (thousands)* ^a^ |  |  |  |  |
| QALY loss illness | 23.2 | 23.0 | 20.4 | 19.1 |
| QALY loss mortality | 409.7 | 408.1 | 386.8 | 370.4 |
| Total QALY loss | 432.9 | 431.1 | 407.2 | 389.4 |
|  |  |  |  |  |
| ***Within children aged 2-16y*** |  |  |  |  |
| *Costs (€, millions)* ^a^ |  |  |  |  |
| Vaccination | 17 | 57 | 229 | 303 |
| Direct HC costs | 65 | 59 | 43 | 39 |
| Indirect HC costs | 0.0 | 0.1 | 0.2 | 0.5 |
| Patient costs | 128 | 126 | 98 | 82 |
| Productivity loss | 179 | 175 | 140 | 115 |
| Total costs | 390 | 416 | 510 | 539 |
| *QALY loss (thousands)* ^a^ |  |  |  |  |
| QALY loss illness | 6.2 | 6.1 | 4.7 | 3.9 |
| QALY loss mortality | 1.1 | 1.0 | 0.9 | 0.8 |
| Total QALY loss | 7.3 | 7.0 | 5.6 | 4.7 |

^a^: Averaged across 1000 simulations.

Table S4: Sensitivity analysis of coverage in healthy children. The table shows the expected costs and QALY losses in absence and presence of childhood influenza vaccination of children aged 2-16 years in the Netherlands over a period of 20 years. Outcomes are shown for the general population and for children aged 2-16 years only. Future costs and QALYs include an annual discount rate of 4% and 1.5%, respectively.

| Outcome | CP | CP + 2-16y at 25% coverage | CP + 2-16y at 50% coverage | CP + 2-16y at 75% coverage |
| --- | --- | --- | --- | --- |
| ***Within the general population*** |  |  |  |  |
| *Costs (€, millions)* ^a^ |  |  |  |  |
| Vaccination | 964 | 1,103 | 1,250 | 1,401 |
| Direct HC costs | 467 | 433 | 398 | 364 |
| Indirect HC costs | - | 157 | 344 | 551 |
| Patient costs | 482 | 440 | 396 | 354 |
| Productivity loss | 2,214 | 2,069 | 1,911 | 1,754 |
| Total costs | 4,127 | 4,202 | 4,299 | 4,423 |
| *QALY loss (thousands)* ^a^ |  |  |  |  |
| QALY loss illness | 23.2 | 21.2 | 19.1 | 17.0 |
| QALY loss mortality | 409.7 | 391.8 | 370.4 | 346.5 |
| Total QALY loss | 432.9 | 413.0 | 389.4 | 363.5 |
|  |  |  |  |  |
| ***Within children aged 2-16y*** |  |  |  |  |
| *Costs (€, millions)* ^a^ |  |  |  |  |
| Vaccination | 17 | 156 | 303 | 454 |
| Direct HC costs | 65 | 51 | 39 | 29 |
| Indirect HC costs | 0.0 | 0 | 0 | 1 |
| Patient costs | 128 | 104 | 82 | 62 |
| Productivity loss | 179 | 146 | 115 | 87 |
| Total costs | 390 | 458 | 539 | 632 |
| *QALY loss (thousands)* ^a^ |  |  |  |  |
| QALY loss illness | 6.2 | 5.0 | 3.9 | 3.0 |
| QALY loss mortality | 1.1 | 1.0 | 0.8 | 0.5 |
| Total QALY loss | 7.3 | 6.0 | 4.7 | 3.5 |

^a^: Averaged across 1000 simulations.

Table S5: The expected cost-effectiveness of childhood influenza vaccination at 50% coverage in the Netherlands over a period of 20 years. Outcomes are shown for the entire population and for children aged 2-16 years only. Future costs and QALYs include an annual discount rate of 4% and 1.5%, respectively.

| Scenario | Total costs  (€, millions) ^a^ | Total QALY loss (thousands)^a^ | Difference in costs (€, millions)^a^ | Difference in QALY loss (thousands)^a^ | ACER (€ per QALY gained)^b^ | ICER (€ per QALY gained)^c^ |
| --- | --- | --- | --- | --- | --- | --- |
| *Within the total population* |  |  |  |  |  |  |
| CP | 4,127 | 432.9 |  |  |  |  |
| CP + 2-3y | 4,150 | 431.1 | 22.3 | -1.86 | 11,993 | Ext. dominance |
| CP + 2-12y | 4,230 | 407.2 | 103 | -25.7 | 3,998 | Ext. dominance |
| CP + 2-16y | 4,299 | 389.4 | 172 | -43.5 | 3,944 | 3,944 |
| *Within children aged 2-16y only* |  |  |  |  |  |  |
| CP | 390 | 7.32 |  |  |  |  |
| CP + 2-3y | 416 | 7.05 | 26.6 | -0.268 | 99,383 | Ext. dominance |
| CP + 2-12y | 510 | 5.60 | 121 | -1.71 | 70,624 | Ext. dominance |
| CP + 2-16y | 539 | 4.71 | 149 | -2.61 | 57,054 | 57,054 |

^a^: Averaged across 1000 simulations. ^b^: The ACER shows the ICER of all childhood vaccination strategies as compared to CP, ^c^: the ICER excludes strategies that are dominated. Dominance occurs when there is a more effective strategy at lower costs (strict dominance) or a more effective strategy at lower ICER (extended dominance). CP: Current program, ACER: Average cost-effectiveness ratio, ICER: Incremental cost-effectiveness ratio, QALY: Quality-adjusted life year

Table S6: The expected cost-effectiveness of childhood influenza vaccination at 50% coverage in the Netherlands over a period of 20 years. Outcomes are shown for the entire population and for children aged 2-16 years only. Future costs and QALYs include an annual discount rate of 4% and 1.5%, respectively.

| Scenario | Total costs  (€, millions) ^a^ | Total QALY loss (thousands)^a^ | Difference in costs (€, millions)^a^ | Difference in QALY loss (thousands)^a^ | ACER (€ per QALY gained)^b^ | ICER (€ per QALY gained)^c^ |
| --- | --- | --- | --- | --- | --- | --- |
| *Within the total population* |  |  |  |  |  |  |
| CP | 4,127 | 432.9 |  |  |  |  |
| CP + 2-16y at 25% coverage | 4,202 | 413.0 | 75 | -20.0 | 3,736 | 3,736 |
| CP + 2-16y at 50% coverage | 4,299 | 389.4 | 172 | -43.5 | 3,944 | 4,120 |
| CP + 2-16y at 75% coverage | 4,423 | 363.5 | 296 | -69.4 | 4,265 | 4,805 |
| *Within children aged 2-16y only* |  |  |  |  |  |  |
| CP | 390 | 7.3 |  |  |  |  |
| CP + 2-16y at 25% coverage | 458 | 6.0 | 68 | -1.3 | 52,017 | 52,017 |
| CP + 2-16y at 50% coverage | 539 | 4.7 | 149 | -2.6 | 57,054 | 62,127 |
| CP + 2-16y at 75% coverage | 632 | 3.5 | 242 | -3.8 | 63,669 | 78,144 |

^a^: Averaged across 1000 simulations, ^b^: The ACER shows the cost-effectiveness of each childhood vaccination strategy as compared to CP, ^c^: the ICER shows the cost-effectiveness for each successive alternative. A scenario is dominated when there is a more effective strategy at lower costs (strict dominance) or a more effective strategy at lower ICER (extended dominance). CP: Current program, ACER: Average cost-effectiveness ratio, ICER: Incremental cost-effectiveness ratio, QALY: Quality-adjusted life year.

A)


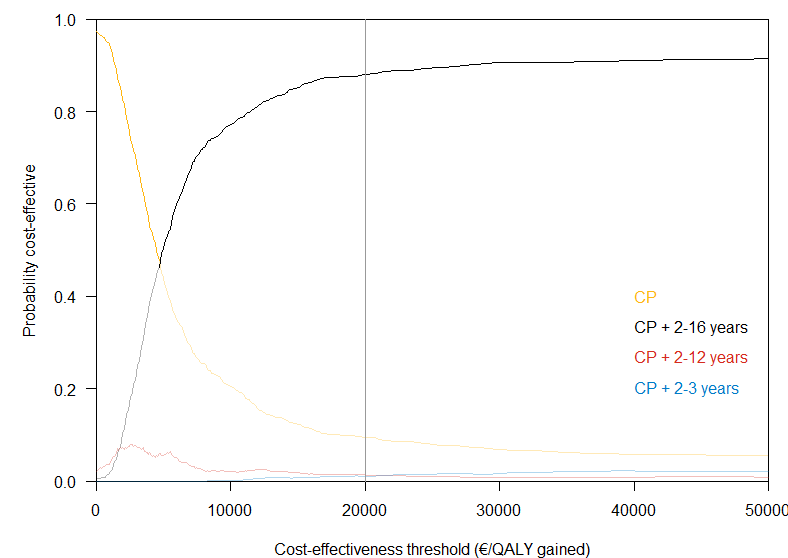
B)


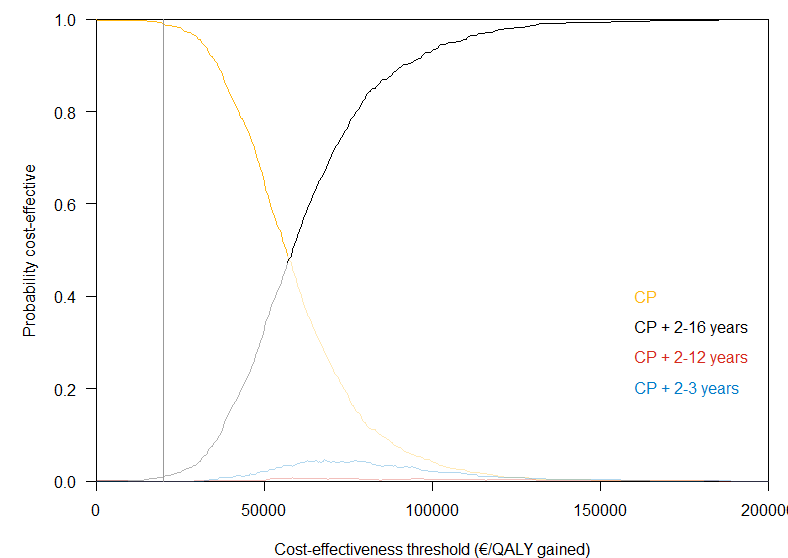


Figure S3: The cost-effectiveness acceptability frontier of childhood influenza vaccination at 50% coverage in the Netherlands over a period of 20 years. The graph shows the most cost-effective alternative over a range of cost-effectiveness thresholds for (A) outcomes in the general population, and (B) outcomes in children aged 2-16 years only. CP: Current program

### Univariate sensitivity analysis of Q-LAIV duration of protection and efficacy

The adapted model is run for a range of vaccination coverages in healthy children, assuming Q-LAIV protects for 2 years with a 50% higher efficacy than TIV. The results for the infection attack rate (IAR) distribution are similar to the results of the original model (Figure S4): as the vaccination coverage increases, the mean and median IAR decrease but the variation increases. However, the median IAR decreases faster in the adapted model results, which effectively increases the variation even further. The age distribution of the mean IAR (Figure S5) shows that the extended and better Q-LAIV has a larger impact on all age classes. The 17-year olds that are not vaccinated, are still protected by the vaccination in the previous year, but a spike in IAR is now observed in the 18-year olds. The simulation with 50% coverage in children is used for the sensitivity analysis in the main text.

The higher impact of Q-LAIV is caused by the longer duration and the higher efficacy. To make a distinction between the two causes, simulations are repeated at 50% coverage, with various Q-LAIV protection durations (1, 2, and 5 years) and various Q-LAIV efficacies (identical to TIV, 50% higher than TIV and inducing full immunity). Table S7 shows that the higher impact of Q-LAIV mainly results from a higher efficacy, and that the duration of protection has limited impact. The reason for this is most probably because of consistent vaccination, where individuals that are vaccinated in one season will also be vaccinated in the next season. As a result, the longer vaccine protection will only be experienced by the 17-year olds that just outgrew the child vaccination programme. We note that even with a vaccine for children that induces full protection for 5 years, the mean infection attack is still considerable and the variation in epidemic size persists.

A)


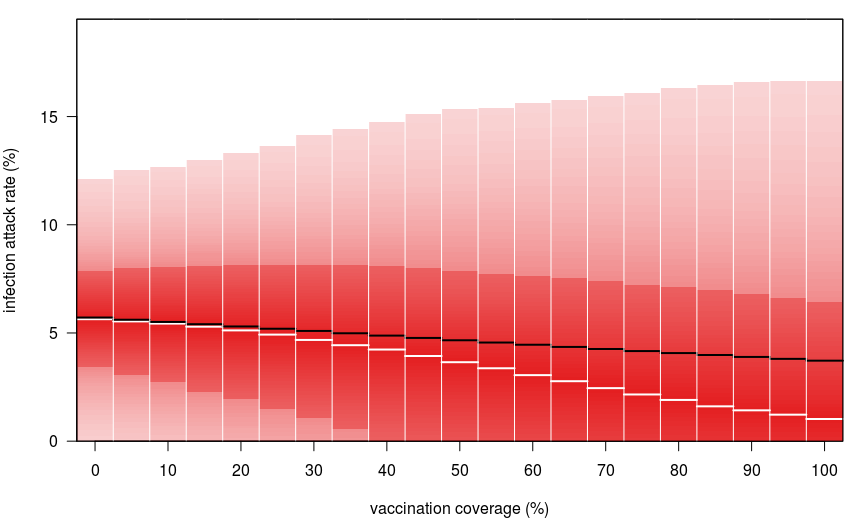


B)


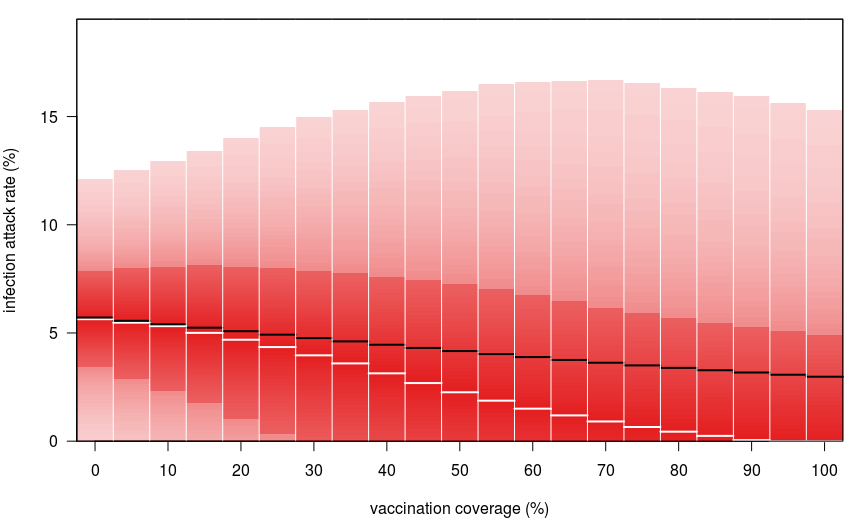


Figure S4: Infection attack rate distribution after roll-out of the paediatric vaccination programme (averaged over the period 2040-2045) for 2-16 year olds as a function of vaccination coverage for the (A) original model, assuming a duration of protection of Q-LAIV of 1 year and vaccine efficacy of Q-LAIV and TIV identical, and the (B) adapted model, assuming duration of protection of Q-LAIV of 2 years and vaccine efficacy of Q-LAIV 50% higher than TIV. The graph shows the mean (black line), median (white line), interquartile range (dark red area) and 95% range (light red area).

A)


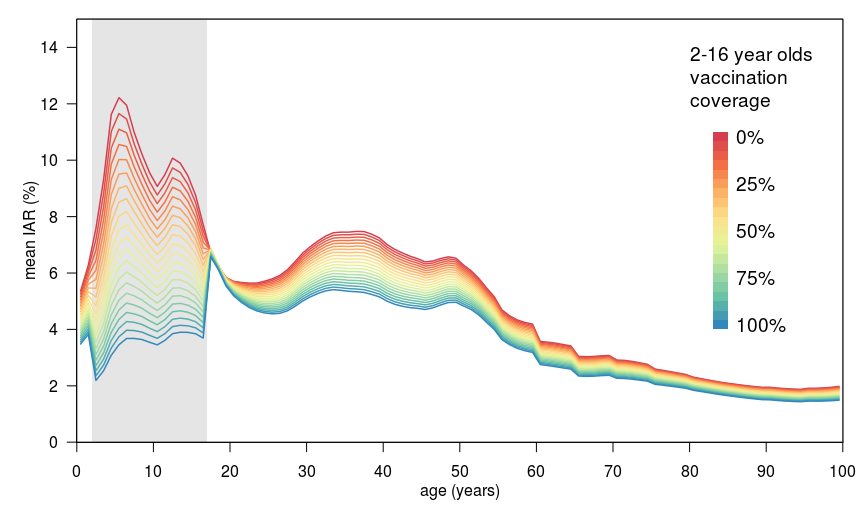


B)


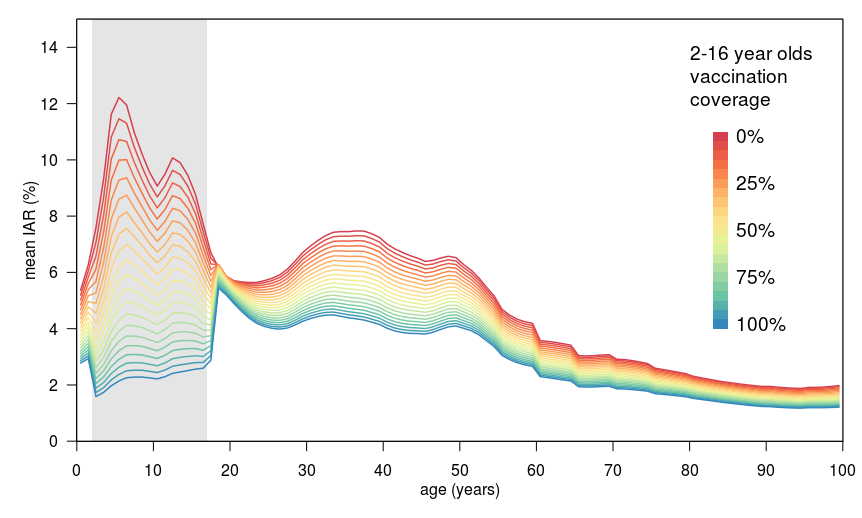


Figure S5: Mean infection attack rate (IAR) per age class after roll-out of the paediatric vaccination programme (averaged over the period 2040-2045) for 2-16 year olds as a function of vaccination coverage for the (A) original model, assuming a duration of protection of Q-LAIV of 1 year and vaccine efficacy of Q-LAIV and TIV identical, and the (B) adapted model, assuming duration of protection of Q-LAIV of 2 years and vaccine efficacy of Q-LAIV 50% higher than TIV. The grey area indicates the age groups targeted in the vaccination programme.

Table S7: Simulated infection attack rate (mean (95% interval)) after roll-out of the paediatric vaccination program (averaged over the period 2040-2045) for 2-16 year olds at 50% coverage, for various characteristics of Q-LAIV.

| Q-LAIV efficacy | Q-LAIV duration | | |
| --- | --- | --- | --- |
|  | 1 year | 2 years | 5 years |
| Identical to TIV | 4.6% (0 – 15.1) ^a^ | 4.6% (0 – 15.3) | 4.5% (0 – 15.5) |
| 50% higher than TIV | 4.3% (0 – 16.0) | 4.2% (0 – 16.2) ^b^ | 4.0% (0 – 16.7) |
| Full protection | 3.7% (0 – 10.8) | 3.5% (0 – 10.7) | 3.2% (0 – 10.3) |

^a^ used in the main analysis, ^b^ explored in economic sensitivity analysis. Q-LAIV: Quadrivalent live-attenuated influenza vaccine, TIV: Trivalent inactivated influenza vaccine

Table S8: Univariate sensitivity analysis of the cost-effectiveness of vaccination of children aged 2-16 years at 50% coverage in the Netherlands.

| Scenario | Base case | Q-LAIV:  2 years protection and 50% higher efficacy | No discounting | Discount rate: 1.5% for costs and health effects | Discount rate: 4% for costs and health effects | Healthcare payer’s perspective | Exclusion of indirect medical costs | Human capital approach | Q-LAIV price of €10.71 | Q-LAIV price of €19.32 | QALY loss Lugner et al. | Halving LE of influenza deaths |
| --- | --- | --- | --- | --- | --- | --- | --- | --- | --- | --- | --- | --- |
| *Difference in costs, (€, millions*)^a^ |  |  |  |  |  |  |  |  |  |  |  |  |
| Vaccination | 286 | 286 | 406 | 353 | 286 | 286 | 286 | 286 | 422 | 586 | 286 | 286 |
| Direct HC costs | -69 | -108 | -98 | -85 | -69 | -69 | -69 | -69 | -69 | -69 | -69 | -69 |
| Indirect HC costs | 344 | 634 | 664 | 510 | 344 | 344 | - | 344 | 344 | 344 | 344 | 191 |
| Patient costs | -86 | -128 | -122 | -106 | -86 | - | -86 | -86 | -86 | -86 | -86 | -86 |
| Productivity loss | -303 | -488 | -430 | -374 | -303 | - | -303 | -346 | -303 | -303 | -303 | -303 |
| Total costs | 172 | 195 | 420 | 298 | 172 | 561 | 172 | 129 | 308 | 472 | 172 | 19 |
| *Difference in QALYs (thousands)*^a^ |  |  |  |  |  |  |  |  |  |  |  |  |
| QALYs illness | -4.2 | -6.2 | -4.8 | -4.2 | -3.4 | -4.2 | -4.2 | -4.2 | -4.2 | -4.2 | -9.2 | -4.2 |
| QALYs mortality | -39.3 | -72.6 | -50.8 | -39.3 | -26.8 | -39.3 | -39.3 | -39.3 | -39.3 | -39.3 | -39.3 | -23.0 |
| Total QALYs | -43.5 | -78.8 | -55.6 | -43.5 | -30.2 | -43.5 | -43.5 | -43.5 | -43.5 | -43.5 | -48.5 | -27.2 |
| *Cost-effectiveness* |  |  |  |  |  |  |  |  |  |  |  |  |
| ICER (€ per QALY gained) | 3,944 | 2,476 | 7,556 | 6,838 | 5,680 | 12,886 | 3,944 | 2,972 | 7,070 | 10,850 | 3,537 | 700 |

^a^: Averaged across 1000 simulations. LE: Life expectancy, QALY: Quality-adjusted life year, Q-LAIV: Quadrivalent live-attenuated influenza vaccine
