# Supplementary material for: Vaccinating children against influenza: overall cost-effective with potential for undesirable outcomes
Source: BMC Med. 2020 Jan 14;18:11. doi: 10.1186/s12916-019-1471-x (PMC6958762; doi:10.1186/s12916-019-1471-x)
Supplement: Supplementary file 3 — Additional file 3. Supplemental simulations on A/H3N2. [file 12916_2019_1471_MOESM3_ESM.docx]

**Additional file 3**

Supplemental simulations on A/H3N2 to:

*Title:* Vaccinating children against influenza: overall cost-effective with potential for undesirable outcomes

Pieter. T. de Boer, Jantien A. Backer, Albert Jan van Hoek, Jacco Wallinga

Centre for Infectious Disease Control, National Institute for Public Health and the Environment, Bilthoven, The Netherlands

## Model results for influenza A/H3N2

In the original analysis, we used a model that captures the susceptibility against the current circulating influenza strain. For any individual, the influenza infection dynamics are described irrespective of the type or subtype of the influenza strain that caused a previous infection; a person becomes susceptible to infection by the circulating strain again after on average 5.1 (95% interval: 2.9 – 8.1) years after the last infection. The waning rate of protection thus captures both the loss of (cross)immunity and the rate of antigenic drift of specific types and subtypes. This approach matches the purpose of the model to study long-term effects of childhood vaccination.

An alternative approach would be to model the infection dynamics for each influenza type and subtype separately. The parametrization of the resulting model will be different. Here we demonstrate that similar outcomes are obtained as those reported in the main text, even when the model reflects only one subtype (influenza A/H3N2) and is fitted to observations for that subtype.

### Parameter estimation

As explained in reference [1], the seasonal influenza attack rate in the Netherlands over the period 2003-2015 was inferred from ILI data from GP’s, using virological data, estimated probabilities to visit the GP and the influenza symptomatic rate. The infection attack rate for influenza A/H3N2 is obtained from this total infection attack rate by multiplication with the proportion influenza A/H3N2 in the circulating strains in each season (see Fig. S4A in [1]), yielding an annual infection attack rate of on average 4.3%, varying from 0.13% to 13% for influenza A/H3N2. The vaccine effectiveness against influenza A/H3N2 is taken from literature as 36 (13 – 61)% (see Fig. S4B in [1]).

Following the same procedure as outlined in [1], the infection attack rate distribution and the vaccine effectiveness distribution are used to estimate the model parameters. The estimated values are presented in Table S1. The average period for a person infected by influenza A/H3N2 to become susceptible again to influenza A/H3N2 infection is 11 (3.8 – 17) years.

Table S1: Estimated parameters (median and 95% credible interval) for influenza A/H3N2 and circulating strain model.

| Parameter | Influenza A/H3N2 | Circulating strains |
| --- | --- | --- |
| R_0_ | 2.0 (1.2 – 2.8) | 1.8 (1.3 – 2.7) |
| Mean waning rate (/year) | 0.087 (0.060 – 0.26) | 0.19 (0.12 – 0.35) |
| Sd waning rate (/year) | 0.017 (0.0055 – 0.045) | 0.031 (0.011 – 0.067) |
| Mean vaccine match | 0.49 (0.37 – 0.60) | 0.56 (0.49 – 0.66) |
| Sd vaccine match | 0.12 (0.086 – 0.14) | 0.11 (0.078 – 0.14) |

### Childhood vaccination

With the estimated parameter values, the effect of the childhood vaccination program on the infection attack rate of influenza A/H3N2 is simulated for a range of vaccination coverages, as was done in [2] and Additional file 2: Figure S4. Figure S1 shows that childhood vaccination increases the variability in epidemic size for influenza A/H3N2. These results demonstrate that similar outcomes are obtained to those reported in the main text, even when the model is applied to types and subtypes separately.


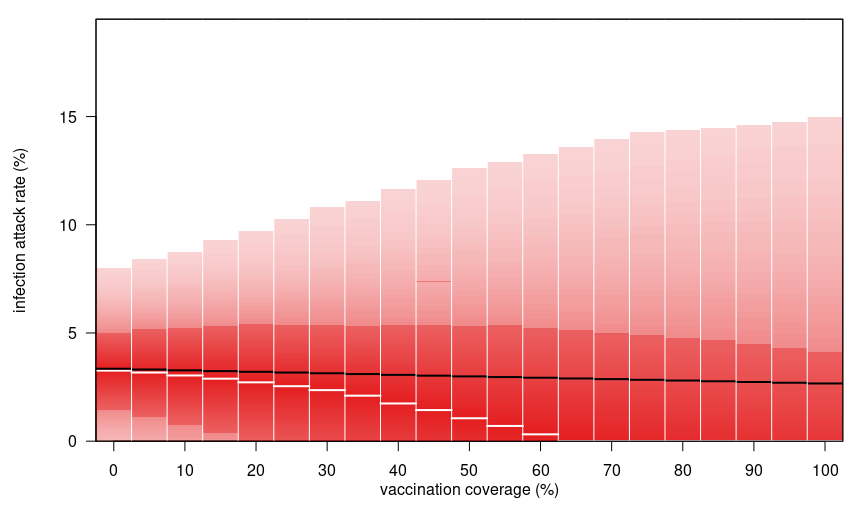


Figure S1: Infection attack rate (IAR) distribution after roll-out of the childhood vaccination program (averaged over the period 2040-2045) for 2-16 year-olds as a function of vaccination coverage for the original (top) and adapted (bottom) model; mean (black line), median (white line), interquartile range (dark red area) and 95% range (light red area).

### Sensitivity to Q-LAIV characteristics

In the original model simulations as well as in the simulations of the previous section, the influenza vaccine used for children was assumed to have the same vaccine efficacy as TIV and provide protection for one year. As in Additional file 2: Table S7, the simulations are repeated at 50% coverage with various Q-LAIV protection durations (1, 2, and 11 years) and various Q-LAIV efficacies (identical to TIV, 50% higher than TIV and inducing full protection). The results in Table S2 reveal similar trends as those reported in the main text: the Q-LAIV duration has limited impact on the infection attack rate of influenza A/H3N2 and the variation in epidemic size persists.

Table S2: Simulated infection attack rate for influenza A/H3N2 (mean (95% interval)) after roll-out of the pediatric vaccination program (averaged over the period 2040-2045) for 2-16 year-olds at 50% coverage, for various characteristics of Q-LAIV

| Q-LAIV efficacy |  | Q-LAIV duration |  |
| --- | --- | --- | --- |
|  | 1 year | 2 years | 11 years |
| Identical to TIV | 2.7% (0 – 11.3)^a^ | 2.7% (0 – 11.2) | 2.5% (0 – 11.2) |
| 50% higher than TIV | 2.6% (0 – 12.9) | 2.6% (0 – 12.9) | 2.4% (0 – 13.0) |
| Inducing full protection | 2.3% (0 – 7.7) | 2.2% (0 – 7.6) | 1.7% (0 – 6.8) |

^a^: used in the simulations of Figure S1

### Number of seasons with large epidemics

Finally, we examine the number of seasons with large epidemics caused by influenza A/H3N2. Based on the observed seasons, a season with a large epidemic is defined as a season with a symptomatic infection attack rate of 4% or higher. We find that in 33% of the simulations childhood vaccination at a 50% coverage leads to an increase in the number of seasons with large epidemics over 20 years. These results demonstrate that similar outcomes are obtained to those reported in the main text, even when the model is applied to types and subtypes separately.

## References

1. Backer JA, Wallinga J, Meijer A, Donker GA, van der Hoek W, van Boven M. The impact of influenza vaccination on infection, hospitalisation and mortality in the Netherlands between 2003 and 2015. Epidemics 2019, 26:77-85.

2. Backer JA, van Boven M, van der Hoek W, Wallinga J. Vaccinating children against influenza increases variability in epidemic size. Epidemics 2019, 26:95-103.
